# Coordination versatility and enhanced bioactivity of Co^2+^, Cu^2+^ and Cd^2+^ complexes derived from pyridyl Schiff base: structural, electronic and electrochemical insights

**DOI:** 10.1186/s13065-026-01730-3

**Published:** 2026-02-21

**Authors:** Abrar K. Elshewemy, Esam A. Gomaa, Hamed M. Abou El Nadar, Abdullah H. Mannaa, Rania R. Zaky, Marwa G. Elghalban, Mahmoud N. Abd El-Hady

**Affiliations:** Department of Chemistry, Faculty of Science, Mansoura University, Mansoura, Egypt

**Keywords:** Pyridine dicarbohydrazide, Schiff base, Cyclic voltammetry, Gaussian, Biological activities

## Abstract

A Schiff base ligand, H_2_L, was obtained from the condensation of pyridine-2,6-dicarbohydrazide and cinnamaldehyde and was further complexed with Co^2+^, Cu^2+^, and Cd^2+^ ions. The coordination complexes were then analyzed by normal spectroscopic/microscopic analyses (FT-IR, UV–visible, ^1^H-NMR, ^13^C-NMR, ESR, PXRD, EDX, and TEM) and other conventional physicochemical analyses (C.H.N microanalysis, molar conductivity, and elemental analysis, molar conductivity) beside (TG/DTA) thermal analysis. Structural analysis revealed mononuclear octahedral geometries for the Co^2+^ and Cd^2+^ chelates through pincer ONO chelation, while the Cu^2+^ complex showed a ligand-supported dinuclear square planer arrangement via two NO hydrazone scaffolds. Computational studies based on the DFT theory postulated the reactivity and spectral trends for the free ligand and metal chelates. The electrochemical properties for the ligand and its complexes were analyzed using cyclic voltammetry and revealed quasi-reversible/irreversible electrochemical behaviors. Biological analyses confirmed the efficiency of the Schiff base and its metal complexes for antibacterial, antifungal, DPPH-antioxidant, and DNA-binding capabilities and exhibited *in-vitro* cytotoxicity towards HepG2 and MCF-7 cell lines.

**Supplementary Information:**

The online version contains supplementary material available at 10.1186/s13065-026-01730-3.

## Introduction

The pyridine-2,6-dicarbohydrazide can act as ONO chelating ligand because its framework contains two functional carbonyl groups supported with central pyridine ring. This moiety enable it to form a stable pincer chelation skeleton [1]. Schiff bases derived from this hydrazide which containing azomethine groups (C=N) are good acknowledged for strong metal coordination ability and wide biological activities [2]. These ligands have shown diverse biological potential involving anti-inflammatory, anticarcinogenic, antifungal, and antibacterial properties which account their importance in bioinorganic and coordination chemistry [3–6]. They are attractive candidates for the construction of multinuclear metal chelates with applications in catalysis and pharmaceuticals as aresult of their structural flexibility and high affinity for metal cations [7, 8].

Transition metal complexes have concerned attraction in medicinal chemistry due to their unique structural and electronic properties which can clearly control the ligand reactivity and biological behavior [9]. The ionic radius of the metal ion and the variations in oxidation state in addition to the coordination geometry can effect on the stability of the ligand and its medicinal performance. In this context, pyridine based Schiff base bearing (−CH=N–NH−CO−C_5_H_4_N−CO–NH−N=CH−) design appear as versatile ligand can generate mononuclear and binuclear metal complexes [10, 11]. Their donor arrangement allows flexible coordination via the amide carbonyl oxygen and the azomethine or pyridine nitrogen, enabling the formation of structurally robust pincer-type chelates whose geometry depends on the coordinated metal ion.

The Cu^2+^, Co^2+^, and Cd^2+^ Schiff base complexes continue to attract interest due to their promising antioxidant, antimicrobial, anticancer, and DNA-binding activities. Cu^2+^ complexes often exhibit redox activity and strong biological interactions due to their adaptable coordination geometry, while Co^2+^ complexes usually adopt octahedral structures with remarkable therapeutic relevance. Cd^2+^ complexes, although less studied, have distinctive coordination behavior and have been shown to have growing bioactivity in several reports. These metal cation control electronic structure and biological response which is essential for the design of new metal based therapeutic candidates [12].

The present study concerns with the synthesis of polydentate Schiff base derived from bis(3-phenylallylidine)pyridine-2,6-dicarbohydrazide and its complexes with Cu^2+^, Co^2+^ and Cd^2+^. The produced chelates were investigated for antimicrobial activity, DNA binding and cytotoxicity against HepG2 and MCF-7 cancer cell lines. Moreover, the DFT calculations were adjusted to optimize the molecular geometry, analyze frontier HOMO/LUMO molecular orbitals, investigate electrostatic potential distribution and elucidate global reactivity description. The work innovates a structurally distinct ligand and metal complexes and provides a correlation between experimental biological data and the quantum chemical parameters highlighting the novelty of binuclear Cu^2+^ scaffold with comparative biological profiling. Generally, it aims to establish structure activity relationships for the Co^2+^, Cu^2+^ and Cd^2+^ chelates with their potential as candidates for future biomedical and coordination chemistry applications.

## Experimental

### Synthesis of the H_2_L ligand and metal complexes

**Scheme 1:**
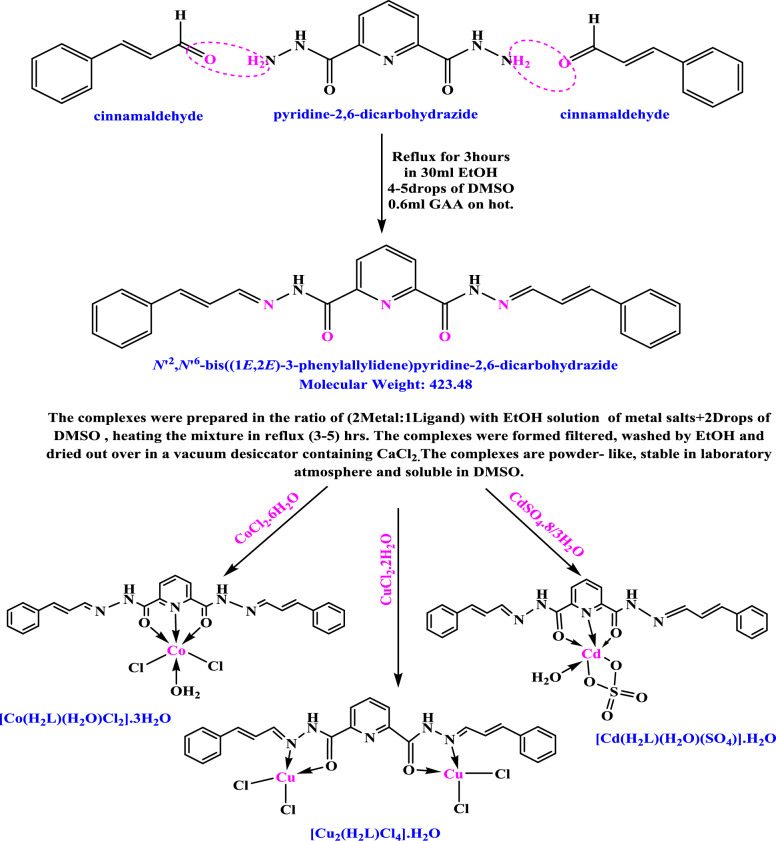
The outline synthesis of ligand H_2_L and its metal complexes

The Schiff base ligand H₂L (bis(3-phenylallylidene)pyridine-2,6-dicarbohydrazide) was synthesized by condensing pyridine-2,6-dicarbohydrazide with cinnamaldehyde in ethanol using glacial acetic acid as a catalyst. The mixture was refluxed for 4 h to afford the crystalline ligand in 80% yield. The metal complexes were prepared by reacting H_2_L with CuCl_2_.2H_2_O, CoCl_2_.6H_2_O, or CdSO₄0.8/3H_2_O in a 1:1 molar ratio under reflux for 4 h. After completion of the reaction, the crude solid was filtered and purified by repeated washing with hot ethanol and distilled water to remove unreacted starting materials and inorganic residues. The ligand was further recrystallized from hot ethanol, while all metal complexes were dried under vacuum over anhydrous CaCl_2_ until constant weight was achieved, ensuring complete removal of residual solvents and obtaining analytically pure products [13, 14]. Synthetic details are illustrated in Scheme 1.

The synthesized ligand and metal complexes were confirmed using IR, NMR, UV–Vis, and mass spectrometry. H_2_L: IR bands at 3171 (NH), 1680 (C=O), and 1626 cm^−1^ (C=N); ^1^H/^13^C NMR signals consistent with the proposed structure; MS: m/z 423.66 [M]⁺; UV–Vis: λmax at 290 and 380 nm. Cu^2+^ complex: IR: 1657 (C=O), 1616 (C=N), 615 (Cu–O), 511 cm^−1^ (Cu–N); UV–Vis: broad band at 13,404 cm^−1^; μeff = 2.64 B.M. Co^2+^ complex: IR: 1651 (C=O), 609 (Co–O), 503 cm^−1^ (Co–N); UV–Vis bands at 15,503 and 19,417 cm^−1^; μeff = 5.17 B.M. Cd^2+^ complex: IR: 1662 (C=O), 614 (Cd–O), 516 (Cd–N); ^1^H/^13^C NMR shifts supporting tridentate coordination.

### Characterization techniques

Elemental analyses (C, H, N) and metal content determinations confirmed the stoichiometry of the isolated compounds. FT-IR, UV–Vis, NMR (^1^H, ^13^C), EPR, and mass spectrometry were employed to characterize the ligand and its complexes. Molar conductivity and Magnetic moment measurements were conducted to evaluate electrolytic and magnetic properties. Thermal behavior was investigated using TG/DTA, while structural and morphological features were analyzed by XRD, TEM, and EDX techniques.

### Electrochemical studies

Cyclic voltammetry (CV) measurements were performed using a DY2100 potentiostat with a conventional three electrode setup consisting of a glassy carbon working electrode (GCWE), an Ag/AgCl reference electrode, and a platinum wire as the counter electrode. The supporting electrolyte solution contained 0.1 M tetrabutylammonium bromide (TBABr) [15, 16], and the concentration of the ligand or metal complex was adjusted to 0.01 M. The overall electrochemical arrangement is illustrated in Scheme 1S.

### Computational methods

DFT calculations (B3LYP/LANL2DZ and 6–311 + G(d,p)) were carried out using Gaussian 09 to optimize geometries, compute HOMO–LUMO energies, and generate MEP maps. The B3LYP hybrid functional was selected due to its well established reliability in modeling transition metal complexes, while the LANL2DZ basis set (with effective core potentials) is appropriate for treating heavy metal centers such as Cu^2+^, Co^2+^, and Cd^2+^. The 6–311 + G(d,p) basis set was used for non metal atoms to ensure accurate geometry and electronic structure predictions. Docking simulations against the 1i7i receptor were performed using MOE 2015 software. From the Protein Data Bank, all proteins were retrieved as PDB files. To avoid interfering with the docking study, water molecules linked to these proteins were removed [17, 18].

### Biological assays

Antimicrobial activity was evaluated via the agar-well diffusion method using *ciprofloxacin* and *clotrimazole* as reference drugs as illustrated in Scheme 2S [19]. Antioxidant activity was assessed using the DPPH radical-scavenging assay as depicted in Scheme 3S, while The *in-vitro* cytotoxic activity against hepatocellular carcinoma (HepG2) and human breast adenocarcinoma (MCF-7) cell lines was evaluated using the MTT assay [20–22] as illustrated in Scheme 4S, with the cell lines obtained from the American Type Culture Collection (ATCC) through the Holding Company for Biological Products and Vaccines (VACSERA), Cairo, Egypt. The DNA binding was screened using the methyl green (MG) assay presented in Scheme 5S [23]. Most of biological procedures were performed in triplicate and the corresponding results are expressed as mean value ± standard deviation (SD). In addition, the statistical significance was assessed where the differences were considered significant at *P* < 0.05.

## Result and discussion

### General characterization

**Elemental analysis**: Microanalysis revealed (C%, H%, and N%) percentages in conjugation with complexometric titrations of metal content (M%) and gravimetric determination of halide/sulfate content (Cl%, SO_4_%). The results showed excellent compatibility with theoretically calculated data, confirming the accuracy of the proposed molecular formulas:[Cu_2_(H_2_L)Cl_4_].H_2_O, [Co(H_2_L)(H_2_O)Cl_2_]0.3H_2_O_,_ and [Cd(H_2_L)(H_2_O)(SO_4_)].H_2_O. It is notable that the cadmium complex is overall electrically neutral where the Cd^2+^ center is exactly balanced by the coordinated sulfato co-ligand (SO₄^2−^) while the Schiff base ligand (H_2_L), the coordinated aqua molecule, and the lattice water molecule are all neutral charged species [17]. On the other side, both the Co^**2+**^ and Cd^**2+**^ complexes incorporated a single metal center per ligand molecule in a 1:1 metal to ligand stoichiometric ratio, resulting in the formation of a mononuclear pincer chelation consist of two fused five membered rings, as depicted in Scheme 1 while the bimetallic Cu^**2+**^ complex exhibited a 2:1 metal to ligand stoichiometry, directing to the production of two separated five membered chelate rings, each coordinated independently to a copper core.

**Molar conductivities:** The molar conductivity of the metal complexes in 10^–3^ M DMSO at room temperature falls in the range (7 and 18 Ω^−1^ cm^2^ mol^−1^) consistent with their non-electrolytic nature of chloro or sulfato complexes. The elemental analysis data beside the physical properties [17] are collected in Table 1. These analytical results provide direct stoichiometric evidence supporting the proposed molecular formulae of the Cu^**2+**^, Co^**2+**^, and Cd^**2+**^ complexes.

**Table 1:** Physical properties and elemental analyses of H_2_L ligand and its complexes

							Elemental analysis found (calculated)				
No.	Compound	M.Wt.	Color		M.p. (^o^C)	Yield%	C%	H%	N%	M%	X%
1	H_2_L	423.48	White		295	80	70.91 (71.86)	5.0 (5.0)	16.54 (16.56)	–	–
2	[Cu_2_(H_2_L)Cl_4_].H_2_O	710.38	Green Olive		>300	70	42.27 (42.52)	3.26 (3.33)	9.86 (9.58)	17.89 (17.67)	19.96 (20.03)
3	[Co(H_2_L)(H_2_O)Cl_2_].3H_2_O	625.37	Pale brown		>300	65	48.02 (48.89)	4.67 (4.31)	11.2 (11.99)	9.42 (10.22)	11.34 (11.58)
4	[Cd(H_2_L)(H_2_O)(SO_4_)**]**.H_2_O	667.97	Pale Yellow		>300	62	44.95 (44.08)	3.77 (3.85)	10.48 (10.6)	16.83 (17.17)	23.96 (24.3)

X = Cl^−^ in Co(II) and Cu(II)-complexes and SO_4_^-^ in Cd(II)-complex, Λ is the molar conductance in μS.mol^−1^.cm^2^. M = Cu (II), Co (II) and Cd(II)

### Spectral studies

**Fig. 1:**
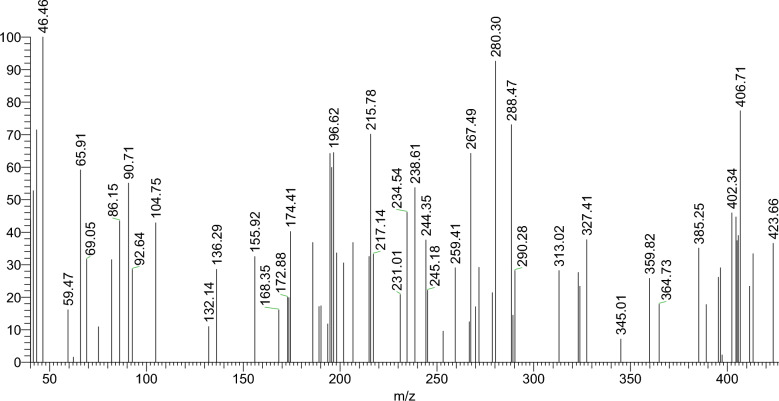
Mass spectrum of ligand

**FT-IR Spectra**: The IR spectrum (Fig. 1S) of the free ligand (H_2_L), displays characteristic absorption bands at 3171, 1680, 1626, and 741 cm^−1^. These correspond respectively to the symmetric stretching of two (NH) groups, the stretching vibrations of two carbonyl (C=O) groups, two azomethine (C=N) groups, and the ring breathing vibration of the pyridine moiety. The IR spectrum of the Cu^**2+**^ complex exhibited lower shifts in the vibration frequencies of ν(C=O) and ν(C=N) to new wavenumbers 1657 and 1616 cm^−1^, respectively, while other groups maintain almost unchanged positions. In addition, it clarifies the manifestation of additional bands at 615 and 511 cm^−1^ which are typical of ν(Cu–O) and ν(Cu–N) [24]. All the previous explanations predict a neutral bidentate chelation from each hydrazone arm to one Cu^**2+**^ center occurs through one carbonyl oxygen atom and another azomethine nitrogen atom forming a binucleating chloro complex. The two aromatic cinnamaldehyde derived arms are oriented and extend outward in opposite directions, minimizing steric congestion and enabling the ligand to accommodate two Cu^**2+**^ ions despite its bulky substituents without requiring a classical *μ*-bridging interaction. Therefore, the dinuclear formulation arises from ligand geometry rather than a shared bridging atom.

On the other side, the IR spectrum of the Co^**2+**^ complex showed displacement of ν(C=O) and pyridine ring breathing vibration to lower wavenumbers 1651 and 751 cm^−1^, respectively. The other functional groups largely retain their positions. As well as the appearance of new bands at 609 and 503 cm^−1^ which are characteristic of ν(Co–O) and ν(Co–N) [25]. All these remarks predict the neutral tridentate pincer chelation of ONO donor ligand via nitrogen of pyridine ring and two carbonyl groups accompanied by one monodentate coordinating water molecule and two coordinating chloro ligand.

Finally, the IR spectrum of the Cd^**2+**^ complex showed a red shift of ν(C = O) to 1662 cm^−1^, and a blue shift of pyridine ring breathing vibration to 747 cm^−1^ without any tangible change in the remaining groups' positions. Further, the appearance of ν(Cd–O) and ν(Cd–N) at 614 and 516 cm^−1^ [25] suggested the neutral tridentate ONO mode of chelation escorted by one monodentate coordinating water molecule and bidentate sulfato group.

Generally, the ligand H_2_L functions as a neutral tridentate ligand coordinating through two carbonyl groups and pyridine nitrogen to the central Co^**2+**^ and Cd^**2+**^ ions, forming pincer type complexes. In contrast, with Cu^**2+**^, H_2_L acts as a neutral tetradentate NOON scaffold, coordinating via two carbonyls and two azomethines. All this data is summarized in Table 2 [26]. These IR coordination shifts are fully consistent with the proposed molecular formulae and the corresponding donor sets assigned for each complex.

**Table 2:** Most important IR bonds of H_2_L ligand and its complexes

Compound	ν(C=O)	ν(NH)	ν(C=N)	ν(M-O)	ν(M-N)	ν(Breathing)_py_
1	1680	3171	1626	–	–	741
2	1657	3160	1616	615	511	746
3	1651	3165	1622	609	503	751
4	1661	3158	1626	614	516	747

**Mass spectrum:** The mass spectrum of the H_2_L ligand demonstrated a molecular ion peak [M]⁺ at m/z = 423.66, which aligns closely with its calculated molar mass. This finding supports the proposed molecular formula, C_25_H_21_O_2_N_5_, with a theoretical molecular weight of 423.48, as shown in (Fig. 1).

**NMR Spectra:** The ^1^H NMR spectrum of the H_2_L ligand predicted sharp singlet peaks at chemical shifts δ 10.66, and 12.19 ppm characteristic for protons of (N**H**) and (N=C−**H**), respectively, as well as multiple peaks at δ (8.12–8.19−8.32) ppm assigned for the protons of the pyridine ring **(C**_**5**_**H**_**3**_**N**). The doublet peaks at δ (7.14−7.17) are marked for the ethylene group protons (**H**−C=C−**H**), and δ (7.40−7.66) belong to the phenyl group protons (C_6_**H**_**5**_). Whereas the ^13^C-NMR spectrum detected carbonyl carbon (**C**=O) as a sharp singlet at δ = 159.38 ppm, azomethine carbon (**C**=N) at δ 139.92 ppm, ethylene carbons (H**C**=**C**H) at δ (135.81−125.60) ppm, pyridine ring carbons (**C**_**5**_H_3_N) in the range δ (139.92 to 124.82) ppm, and phenyl carbons (**C**_6_H_5_) in the range δ (128.93 to 127.28) ppm.

**Fig. 2:**
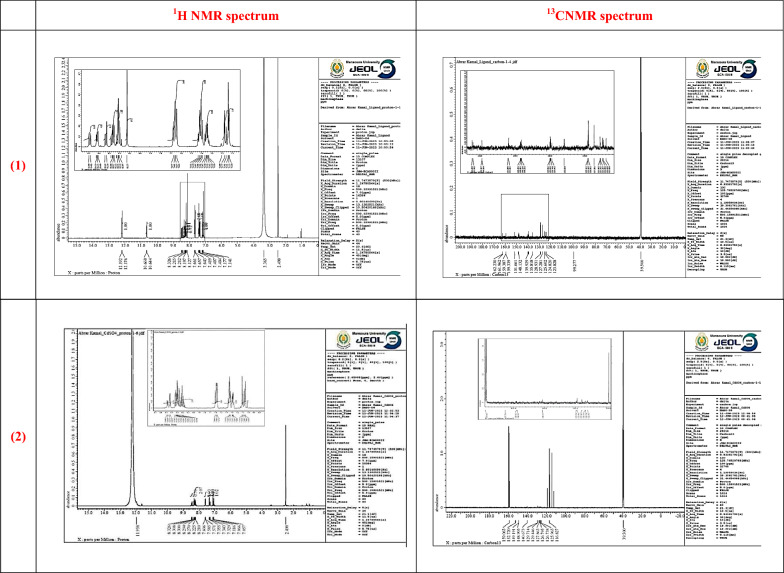
NMR and ^13^CNMR spectra of (1) ligand (H_2_L) and (2) [Cd(H_2_L)(H_2_O)(SO_4_)].H_2_O complex

The ^1^H-NMR spectrum of the Cd^**2+**^ complex predicted sharp singlet peaks at chemical shifts δ 11.6, and 12.00 ppm characteristic for (N**H**) and (N=C−**H**) protons, respectively, as well as multiple peaks at δ (8.19−8.42−8.48) ppm assigned for the protons of the pyridine ring (C_5_**H**_3_N). The doublet peaks at δ (7.05−7.11) are marked for the ethylene group protons (**H**−C=C−**H**), and δ (7.28−7.35) belong to the phenyl group protons (C_6_**H**_**5**_). Whereas the ^13^C-NMR spectrum detected carbonyl carbon (**C**=O) as a sharp singlet at δ = 159.06 ppm, azomethine carbon (**C**=N) at δ 140.67 ppm, ethylene carbons (H**C**=**C**H) at δ (125.96—129.44) ppm, pyridine ring carbons (**C**_**5**_H_3_N) in the range δ (116.82 to 129.71) ppm, and phenyl carbons (**C**_6_H_5_) in the range δ (126.79 to 127.86) ppm. This data is summarized in (Fig. 2) and Table 1S. Hence, the data of NMR analyses confirm the neutral behavior of the ligand in its coordination with Cd^**2+**^ without any change in the proton environment [27].

Compared with the free ligand, the Cd^**2+**^ complex exhibits only slight downfield shifts in the azomethine and carbonyl resonances, while aromatic and aliphatic regions remain largely unchanged. Such minimal changes are characteristic of coordination to a diamagnetic d^10^ metal center such as Cd^2+^, which perturbs the electronic environment only weakly. Nevertheless, the observed deshielding of the (C=N) and (C=O) carbons is consistent with their involvement in metal binding, supporting coordination through the ONO donor set while confirming that the overall ligand framework remains electronically preserved upon complexation.

### ESR, UV–visible and magnetic moment measurements

**Electron spin resonance**: The solid state ESR spectrum of the Cu^2+^ chelate yielded valuable insights into the stereochemistry, metallic-ligand binding surroundings, and magnetic interactions within the complex. The spin Hamiltonian parameters have been determined for [Cu_2_(H_2_L)Cl_4_].H_2_O, where the nuclear spin (I) is 3/2 and the electron spin (S) is 1/2. The ESR spectrum (Fig. 2S) confirmed characteristics of axial symmetry, with G tensor values satisfying condition g_||_> g_⊥_ > 2.0023, and pattern is indicative of the d_x2-y2_ ground state of Cu^2+^, that's aligned with a square planar geometry [28].

The axial ESR parameters of the Cu^2+^ complex (g_∥_ = 2.26 and g_⊥_ = 2.08) yield an exchange interaction parameter of G = 3.2. Although values of G < 4 are sometimes interpreted as suggesting possible exchange interactions in Cu^2+^systems [29]. This interpretation must be made with caution where the slight deviation from the ideal value in the present case, is attributed to normal anisotropic and orbital contributions rather than to true Cu-Cu magnetic exchange. This conclusion is in full agreement with the DFT-optimized geometry, which shows no structural pathway that would enable Cu-Cu superexchange and confirms that each Cu^2+^ ion is coordinated independently by one hydrazone arm of the ligand.

The ratio g_||_/A_||,_ indicator for assessing the degree of such distortion. For square planar Cu^2+^complexes, this ratio typically falls within the range of 105 to 135, although it can range depending on the nature of the donor atoms involved in coordination. In this observe, a value of 129 changed into received, that is consistent with a square planar geometry [30].

The molecular orbital coefficients α^2^ and β^2^ had been calculated using installed empirical equations [31], which describe the degree of covalency inside the bonding interactions between the copper(II) 3d orbitals and the ligand orbitals. Specifically, α^2^ quantifies the in-plane σ-bonding man or woman, even as β^2^ relates to the in-plane π-bonding covalency, as follow equations:

1 $$\alpha^{2} = [(A_{\parallel}/0.036) + (g_{\parallel} -- 2.0023) + 3] / [7(g_{\perp} -- 2.0023) + 0.04]		(1)$$

2 $$\beta^{2} = {\mkern 1mu} \left[ {\left( {{\mathrm{g}}_{{||}} - 2.0023} \right){\text{ E}}} \right]{\mkern 1mu} /\left[ { - 8\uplambda \upalpha ^{2} } \right] $$

where E is the strength of the relevant electronic transition, and λ = -828 cm^−1^ is the spin–orbit coupling regular for a free Cu^2+^ ion. A value of α^2^ = 1 corresponds to purely ionic bonding, whereas α^2^ = 0.5 reflects complete covalent individual, below the assumption of minimum orbital overlap. A decrease in β^2^ suggests an increase within the covalent individual of in-aircraft π-bonding. For the complex beneath investigation, the calculated values had been α^2^ = 0.81 and β^2^ = 0.70, indicating great covalent person, particularly in the in-plane π-bonding interactions. This is regular with the presence of ligand orbitals able to effective overlap with the copper dxy orbital. Moreover, for copper(II) complexes adopting a square planar geometry, a lower β^2^ cost relative to α^2^ indicates that the π-bonding interactions showcase more covalency than the corresponding σ-bonding [32].

**Fig. 3:**
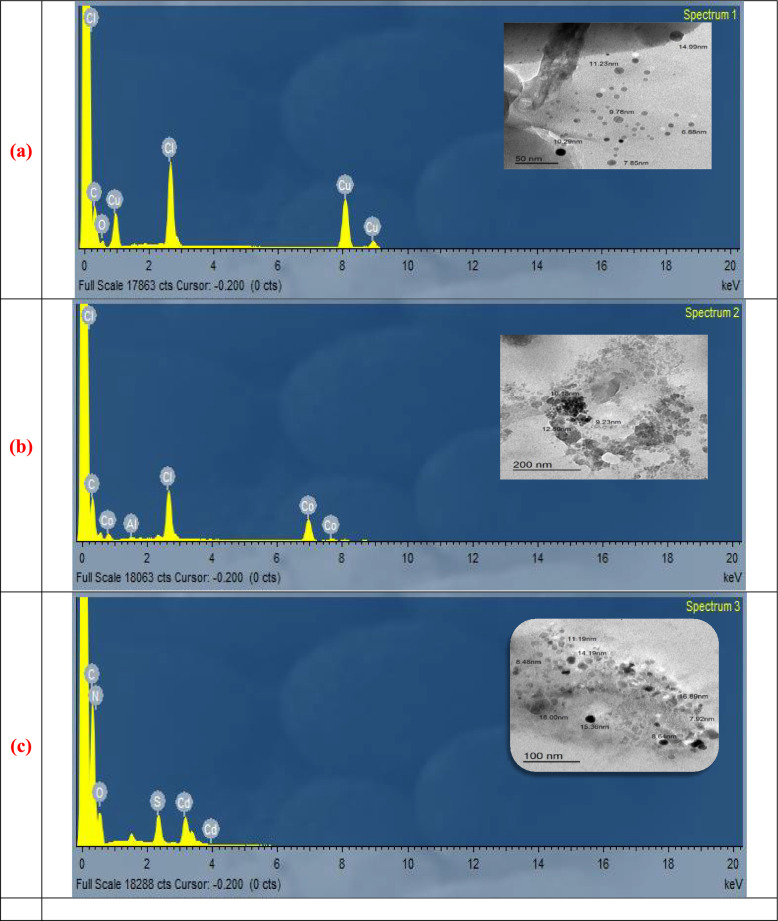
TEM and EDX of **a** [Cu_2_(H_2_L)Cl_4_].H_2_O, **b** [Co(H_2_L)(H_2_O)Cl_2_].3H_2_O and **c** [Cd(H_2_L)(H_2_O)(SO_4)_].H_2_O complexes

**Ulta violet-visible spectra:** The UV–visible spectrum (Fig. 3S) of the free ligand exhibited two prominent absorption bands: the first at 290 nm, assigned to a π → π* transition within the aromatic chromophore, and the second at 380 nm, corresponding to an n → π* transition arising from the non-bonding electrons of the pyridine nitrogen and carbonyl oxygen atoms. In contrast, the UV–visible spectrum of the Cu^2+^ complex displayed a broad d-d transition centered at 13,404 cm^−1^, distinguishing of three spin-allowed transitions (^2^B₁g → ^2^A₁g, ^2^B₁g → ^2^B₂g, and ^2^B₁g → ^2^E₉), which are consistent with a square planar coordination geometry around the Cu^2+^ ion [33].

For the Co^2+^ chelate, the electronic absorption spectrum exhibits two prominent bands at 15,503 and 19,417 cm^−1^. According to the Tanabe-Sugano diagram for a high spin d⁷ system, the ground state of Co^2+^ in an octahedral ligand field is ^4^ T₁g(F). The observed bands are therefore assigned to the spin allowed transitions ^4^ T₁g(F) → ^4^A₂g(F) and ^4^ T₁g(F) → ^4^ T₁g(P), respectively, which are characteristic of octahedral Co(II) complexes with moderate crystal field strength. The corresponding spectroscopic parameters were determined as follows: crystal field splitting energy (Dq) = 836.56 cm^−1^, Racah interelectronic repulsion parameter (B) = 880.59 cm^−1^, nephelauxetic ratio (β) = 0.91, and transition ratio (ν₂/ν₁) = 2.148. These values, along with the calculated first transition state (ν₁ = 7217 cm^−1^), are in excellent agreement with Tanabe-Sugano predictions for distorted octahedral Co^2+^ complexes, supporting the proposed coordination geometry [34].

**Magnetic moment measurements**: The observed magnetic moment of the Cu^2+^ complex (μ-eff = 2.64 B.M.) is slightly higher than the spin only value calculated for two no- interacting Cu^2+^ ions (≈ 2.45 B.M.). This suggests that the two Cu^2+^ centers behave essentially as magnetically independent S = 1/2 units, with only a minor deviation attributable to orbital contributions and experimental uncertainty, rather than to significant ferromagnetic or antiferromagnetic exchange. Furthermore, the experimentally observed magnetic moment (5.17 B.M.) is consistent with a high spin octahedral Co^2+^ chelate [35, 36]. The electronic transitions and magnetic properties further corroborate the proposed formulations of complexes.

### Morphological examinations

**Transmission electron microscopy**: TEM was employed to further characterize the metal complexes where this technique is highly effective for obtaining direct and reliable information regarding the surface morphology and particle size. The TEM micrographs with scale bars (200, 100, and 50 nm) discovered the samples don’t form separate nanoparticles but they appear as nano aggregated crystallites resulting from the self assembly of the molecular complexes. The measured size domains ranged from (6.88 to 14.99 nm for the Cu^2+^, 9.23 to 12.8 nm for the Co^2+^, and 7.92 to 16.89 nm for the Cd^2+^) [37]. These observations point that the nanoscale characteristic grows from aggregation morphology instead of the formation of actual nanoparticles.

**Energy dispersive X-ray**: EDX analysis was used to support the elemental composition of the complex which display the appearance of (C, N, S, O and Cl) together the metal content (Cu, Co, Cd) without any impurity or foreign elements which confirming the purity of the synthesized compounds. The carbon peaks are attributed to the investigated organic moiety. Additionally, the experimentally estimated elemental percents are closely matched with the theoretical values, further validating the successful synthesis of the target metal complexes as shown in Fig. 3 [38, 39].

**Fig. 4:**
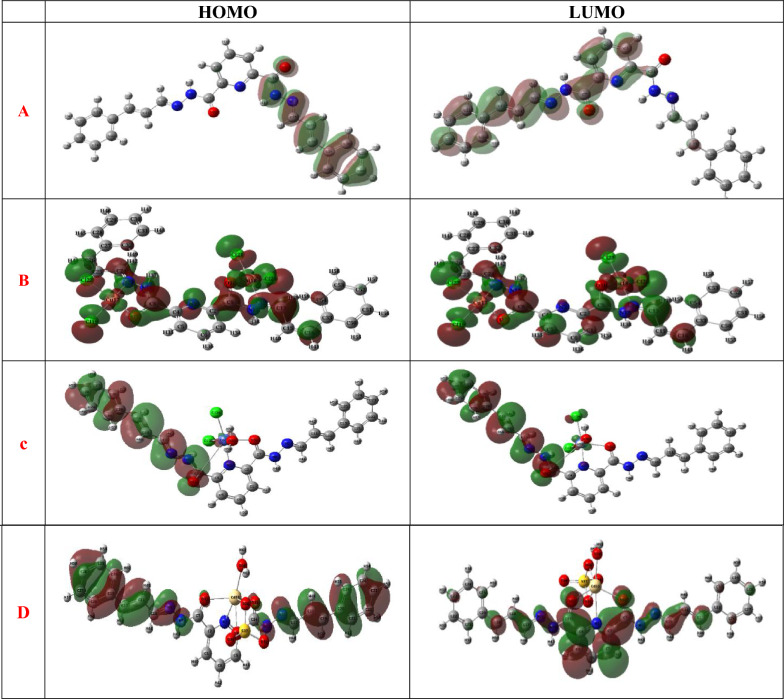
The HOMO and LUMO of **A** H_2_L, **B** [Cu_2_(H_2_L)Cl_4_].H_2_O, **C** [Co(H_2_L)(H_2_O)Cl_2_].3H_2_O and **D** of [Cd(H_2_L)(H_2_O)(SO_4)_].H_2_O complexes

**Powder X-ray diffraction**: The PXRD was achieved at room temperature using Cu Kα radiation (λ = 1.5418 Å) within the 2θ range of 10°-80°. The diffraction patterns obtained for the Cu^2+^ and Cd^2+^ complexes as shown in Fig. 4S, propose a semi-crystalline nature. This behavior indicate the existence of nanocrystalline domains in the aggregated structures [40]. Key diffraction parameters including diffraction angle (θ = 11.08°, 13.62°), inter-planar spacing (d = 4.01 Å, 3.27 Å), and peak broadening (FWHM = 0.10) at a relative intensity (I = 100%) were determined. The average crystallite size (D) derived from Debye–Scherrer and Bragg equations (Eqs. 3 and 4), were estimated (80.92 nm and 81.71 nm). In addition, the dislocation density (δ = 1/D^2^) and microstrain (ε = β/4tanθ) were evaluated where the calculated δ values were found to be in the order of 1.5 × 10^–4^ nm^−2^ while the microstrain ε varied between (1.8–2.2 × 10^–3^), informing low lattice strain and a moderate defect density within the nanocrystalline domains.

3 $${\beta} \, = \, 0.{9 }\uplambda \, /{\text{ S}} \cos \left( \uptheta \right) $$

4 $$ {\text{n }}\uplambda = {\text{ 2 d}} \sin \left( \uptheta \right)\quad {\text{at n}} = {1} $$

### Thermal analysis

Thermal estimation was used to estimate the stability of the prepared metal complexes, predict the degradation pathways and confirm the proposed formulas [Cu_2_(H_2_L)Cl_4_].H_2_O, [Co(H_2_L)(H_2_O)Cl_2_]0.3H_2_O, [Cd(H_2_L)(H_2_O)(SO_4_)].H_2_O.

This technique additionally supplied insights into the presence and nature of coordinated and uncoordinated water molecules, the position of anions (within or outside the coordination sphere), and the series of ligand decomposition. Thermogravimetric (TGA) and differential thermal evaluation (DTA) have been done under a nitrogen environment from ambient temperature up to 800 °C. The decomposition profiles and corresponding mass losses for the chelates are summarized in Table 2S, showing strong settlement among experimental and theoretical weight reduction values.

**Fig. 5:**
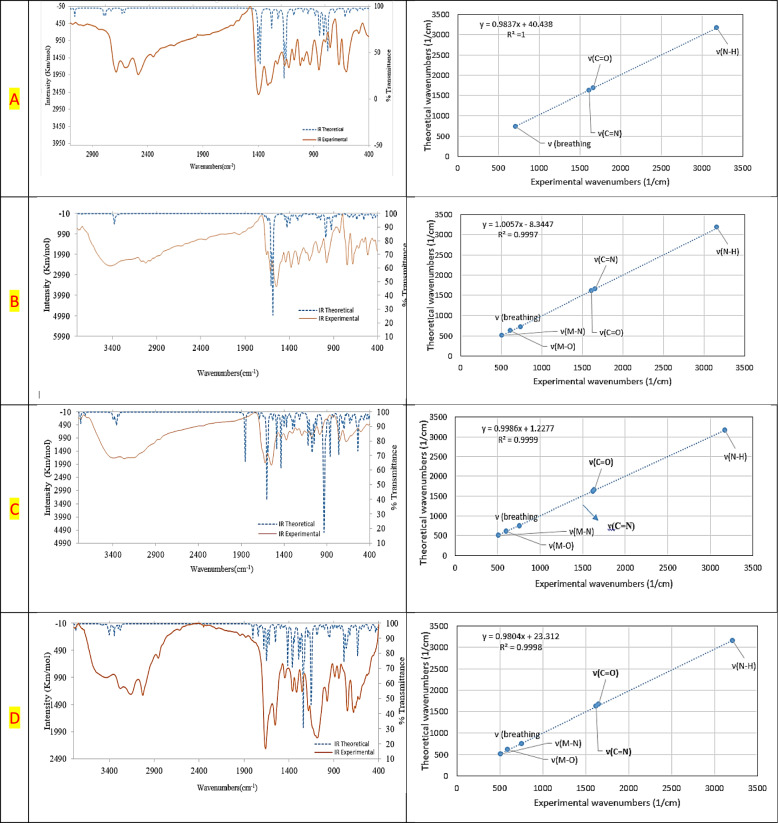
Converged frequencies and scatter plots of experimental versus theoretical FTIR of **A** H_2_L, **B** [Cu_2_(H_2_L)Cl_4_].H_2_O, **C** [Co(H_2_L)(H_2_O)Cl_2_].3H_2_O and **D** of [Cd(H_2_L)(H_2_O)(SO_4_)].H_2_O complexes

The TGA curve of the copper(II) chelate (Fig. 5S) confirmed an initial mass loss of 2.20% in the temperature range of 22–124 °C, which corresponds to the discharge of molecule of lattice (hydrated) water. This changed into followed by way of a significant weight loss of 32.72% between 125–418 °C, attributed to the removal of chlorine molecules and pyridine (C_5_H_5_N) fragments. A similarly 21.38% mass loss become located from 419–594 °C, which can be ascribed to the lack of 2 benzene rings (C_6_H_6_). The final predominant decomposition step befell between 595–665 °C, accounting for a 21.45% weight reduction and regarding degradation of the ultimate organic framework (C_8_H_4_N_4_) and disruption of the chelate structure. The residual mass (22.22%) changed into steady with the formation of 2CuO components, in accordance with the theoretical value (Scheme 6S).

The cobalt(II) chelate (Fig. 5S) displayed an preliminary weight loss of 11.37% within the temperature region of 22–206 °C, similar to the loss of 3 hydrated water molecules and the departure of coordinated water molecule [41]. In the temperature region of 207–299 °C, a 12.16% weight loss became discovered, possibly because of the removal of two chloride atoms. Subsequent decomposition of the ligand's natural framework happened in predominant steps: 44.86% loss from 300–416 °C, attributed to 2 C_9_H_9_N_2_ components, and 19.43% from 417–470 °C, associated with a C_7_H_3_NO moiety. The closing residue, accounting for 12.16% of the preliminary mass, corresponded to CoO, in settlement with theoretical expectancies (Scheme 7S).

The cadmium (II) chelate (Fig. 5S) confirmed a preliminary weight reduction of 5.49% among 22–207 °C, corresponding to the mass reduction of one hydrated water molecule and another coordinating aqua ligand. A giant decomposition step happened from 208–367 °C, with a 38.39% loss attributed to the elimination of SO_2_, O_2_, and two benzene ring (C_6_H_6_). Between 368–538 °C, the complicated lost an extra 25.07% of its mass, which corresponds to the degradation of the (C_8_H_8_N_4_O) ligand fragment. In the very last level, from 53 to 599 °C, a 12.22% weight loss becomes recorded, associated with the partial breakdown of a final ligand portion and the discharge of an uncoordinated pyridine moiety (C_5_H_3_N). The very last residue over 599 °C is turned into CdO, comprising 18.81% of the unique mass, in step with calculated values [42] (Scheme 8S).

### Computational studies based on DFT

**Fig. 6:**
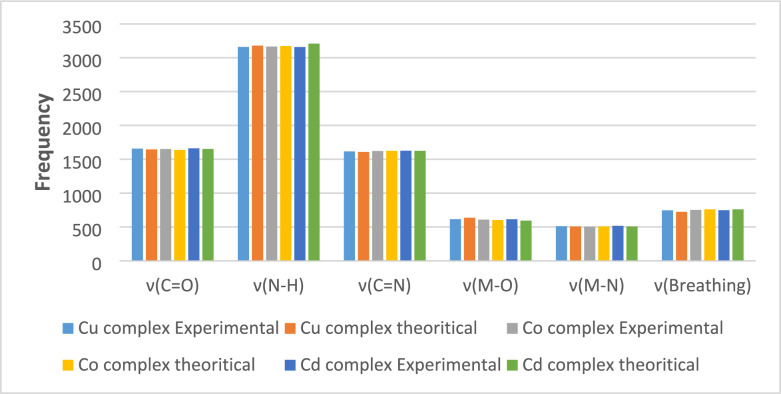
Comparison of theoretical and experimental FTIR frequencies

**Frontier Molecular Orbitals**: The optimized geometries generated from the DFT calculations (Fig. 6S), including atom numbering, show excellent consistency with the experimentally established molecular formulae of the complexes. Additionally, the dipole moment and the overall energy for every structure have been evaluated [43]. Key electronic structure parameters, which includes the lowest unoccupied molecular orbital (E_LUMO_), highest occupied molecular orbital (E_HOMO_), and power hole (Egap), have been computed. These parameters have been then utilized to derive various molecular chemical descriptors, together with μ (chemical potential), χ (electronegativity), η − (hardness), and ω (global electrophilicity index), using the subsequent Eqs. (5–10) [44] as outlined in Table 3.

5 $$ {\mathrm{E}}_{{{\mathrm{gap}}}} = {\text{ E}}_{{\mathrm{(LUMO)}}} - {\mathrm{E}}_{{\mathrm{(HOMO)}}} $$

6 $$ \upeta^{ - } = {\text{ E}}\left[ {_{{({\mathrm{LUMO}})}} - {\text{ E}}_{{({\mathrm{HOMO}})}} } \right] \, /{2} $$

7 $$\omega \, = \, \upmu ^{2}/{2}\upeta $$

8 $$ \upmu \, = \, - \, \upchi \, = {\text{ E}}_{{({\mathrm{LUMO}})}} + {\text{ E}}_{{({\mathrm{HOMO}})}} /{2} $$

9 $$ \upchi \, = \, - {\text{ E }}\left[ {_{{({\mathrm{LUMO}})}} + {\text{ E}}_{{({\mathrm{HOMO}})}} } \right] \, /{2} $$

10 $$ \upsigma = { 1 }/\upeta^{ - } $$

**Table 3:** Electronic energy, Dipole moment and calculated data from energies of E_HOMO_ and E_LUMO_ for H_2_L and its complexes

Compounds	Electronic energy (Ha)	Dipole moment (De)	E_HOMO_ (eV)	E_LUMO_(eV)	E_gap_(eV)	η (eV)	σ (eV)	μ (eV)	χ (eV)	ω (eV)
1	− 1389	6.729	− 0.2155	− 0.089	0.126	0.0633	15.80	− 0.152	0.152	0.183
2	− 1840	12.876	− 0.1858	− 0.171	0.014	0.0073	136.9	− 0.178	0.178	2.183
3	− 1640	12.422	− 0.2115	− 0.117	0.093	0.0469	21.32	− 0.164	0.164	0.288
4	− 1823	12.213	− 0.2117	− 0.128	0.083	0.0416	24.03	− 0.170	0.170	0.347

The outcomes of the DFT calculations cause the subsequent conclusions: (i) The negative values of the frontier molecular orbital energies (HOMO and LUMO) (Fig. 4), along with the positive HOMO–LUMO energy gaps obtained from DFT calculations, confirm that the ligand and its metal complexes possess stable ground states. (ii) The copper chelate exhibited the smallest electricity gap (Egap = 0.0148 eV), which means increased polarizability and reactivity, probably improving its biological activity. (iii) The assignments of the ν(M–O) and ν(M–N) bands in the 500–600 cm^−1^ region are further supported by the DFT-optimized geometries, which show clear shortening of the M–O and M–N bond lengths relative to the free ligand. The calculated M–O distances (1.915–2.360 Å) and M–N distances (1.930–2.493 Å) fall within the typical coordination ranges reported for Cu^2+^, Co^2+^, and Cd^2+^ complexes as shown in Table 3S, providing computational confirmation of the coordination through carbonyl oxygen and azomethine/pyridyl nitrogen atoms. These structural parameters validate the IR based assignments and reinforce the proposed coordination mode. (iv) The bond angles represented in Table 4S propose that the [Cu_2_(H_2_L)Cl_4_].H_2_O complex adopts a square planar geometry, whilst the [Co(H_2_L)(H_2_O)Cl_2_]·3H_2_O and [Cd(H_2_L)(H_2_O)(SO_4_)].H_2_O chelates are steady with an octahedral geometry. These findings are in keeping with the experimental UV–visible spectral information. (v) The ligand (H_2_L) displayed a lower electrophilicity index (ω = 0.1831 eV) compared to its metal chelates, which exhibit better values. The ligand additionally confirmed the best chemical potential (μ = -0.15224 eV) and the bottom electronegativity (χ = 0.15224 eV), which can be linked to better antioxidant interest. This trend is steady with the concept that electrophilicity decreases as the chemical potential will increase [45].

**Table 4:** The outcome data of H_2_L docking with the 1i7i protein receptor

Compound	Receptor amino acid	S	rmsd	No. of bonds	Total energy
1	H-acceptor Ionic pi-H	− 8.5413	1.455	7	28.2
2	H- acceptor	− 6.9126	1.877	3	− 7.9
3	H-donor H- acceptor pi-H	− 7.1054	1.9553	5	− 10.6
4	H-donor H- acceptor	− 7.8873	1.5344	3	− 9.7

**Validation of the calculated vibrational frequencies:** A statistical comparison was performed between experimentally measured FTIR frequencies and DFT-calculated values for the ligand and it's Cu^2+^, Co^2+^ and Cd^2+^ complexes. Theoretical IR frequencies were obtained from DFT calculations as described (B3LYP functional with LANL2DZ/6–311 + G(d,p) basis set, gas phase model and standard Gaussian convergence criterion). Correlation plots including convergence frequencies and scatter plots (Fig. 5) show that the calculated vibrational modes closely match the experimental ones, with the data points located close to the regression line and showing high coefficients of determination (R^2^). The extracted R2 values were 0.94 for the free ligand, 0.9979 for the Cu^2+^ complex, 0.9999 for the Co^2+^ complex, and 0.9780 for the Cd^2+^ complex, confirming the excellent linear correlation across all systems. This strong linear relationship indicates that the computational model reliably reproduces the observed spectral features. Minor deviations observed in the plots (Fig. 6) fall within normal experimental uncertainty and reflect expected limitations of the harmonic approximation used in DFT. Overall; the statistical agreement between experimental and theoretical FTIR data confirms the validity of the computational approach and supports the vibrational assignments made for the coordination compounds.

**Fig. 7:**
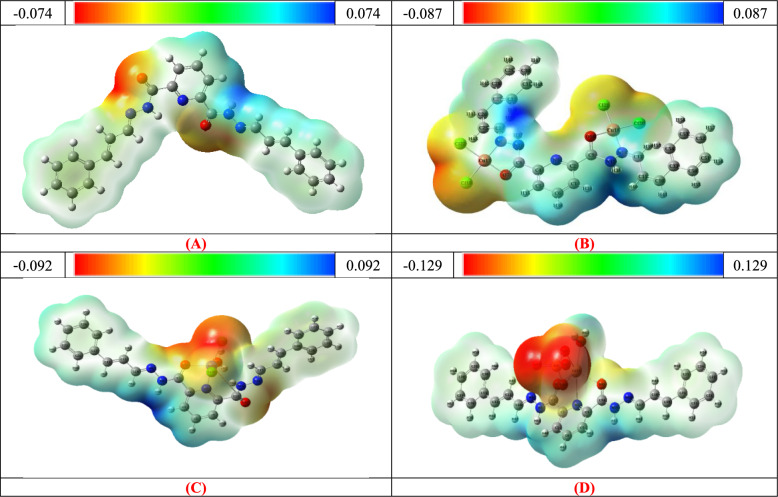
Molecular electrostatic potential map for **A** H_2_L, **B** [Cu_2_(H_2_L)Cl_4_].H_2_O, **C** [Co(H_2_L)(H_2_O)Cl_2_].3H_2_O and **D** [Cd(H_2_L)(H_2_O)(SO_4)_].H_2_O complexes

**Electrostatic potential distribution:** The molecular electrostatic potential (MEP) distributions for the matching metallic chelates and the H_2_L ligand are shown in Fig. 7. There are three different zones on the electrostatic potential map. The blue area indicates regions with low electron density, which may be associated with sites that are vulnerable to nucleophilic attacks. regions with a neutral electrostatic potential are represented by the green region, whereas regions with a high electron density a sign of electrophilic attack sites are represented by the red area [46]. The investigation shows it clear that the negative electrostatic potential is primarily concentrated around electronegative atoms, such as the chloride ions and oxygen atoms in the carbonyl and sulfate groups. On the other hand, electropositive atoms particularly the hydrogen atoms in the amide groups are the center of positive electrostatic potential.

The FMO results indicate that the Cu(II) complex, with the smallest HOMO–LUMO gap, is the most chemically reactive species, which is consistent with its electrochemical behavior. This correlation is consistent with the CV results, as complexes with shorter HOMO–LUMO gaps generally undergo electron transfer at a more accessible potential. The ligand shows the greatest chemical potential and the smallest electrophilicity index, connected to its high antioxidant and cytotoxic impact. MEPs show that the carbonyl oxygen, sulfate oxygen, and azomethine nitrogen act as nucleophilic active sites, while NH and aromatic hydrogen represent electrophilic areas. In common, the findings establish a straight connection between the electronic structure and the obtained biological screening.

### Biological assessment

**Microbiological evaluation:** The antimicrobial activity of the metal chelates can be explained by the chelation theory which states that the chelation process reduces the polarity on the metal ion by assist possible delocalization of π-electrons in the chelate ring as well as partial delocalization of its positive charge on the donor atoms. This in turn raises the lipophilicity of the chelate facilitating its interaction with microbial cell membrane. The Activity index (AI) and Minimum inhibitory concentrations (MIC**)** values were measured to complement the inhibition zone results (Table 5S). AI represents the ratio of the diameter of inhibition zone diameter for the tested compound to that of the corresponding standard antimicrobial agent [47]. The compounds were tested *in-vitro* against *B.subtilis* (Gram + ve bacterial strain), *E. coli* (Gram-ve baccterial strain) and *C. albicans* fungus using *ciprofloxacin* and *clotrimazole* references.

**Table 5:** Cyclic voltammetry of H_2_L ligand and its complexes

Compound	Ep,a Volt	Ep,c Volt	∆Ep Volt	(−) Ip,a × 10^6^ Amp	Ip,c x10^6^ Amp	Ip,a/Ip,c	E˚ Volt
1 (Peak one)	− 0.0276	− 0.3913	0.3637	6.00	2.56	2.3458	− 0.21
1 (Peak two)	–	− 0.6596	0.6596	–	7.92	0	− 0.33
2	0.5045	0.4237	0.0807	1.29	2.82	0.4554	0.4641
3	–	− 0.6784	0.6784	–	7.13	0	− 0.339
4	0.5851	− 0.6637	1.2488	3.93	5.97	0.6579	− 0.039

**Fig. 8:**
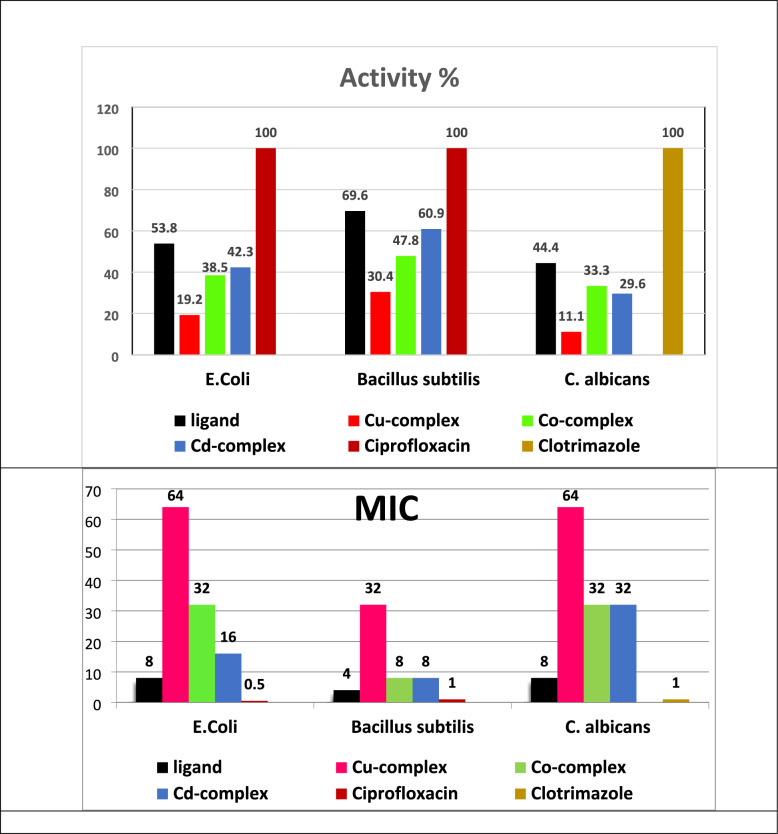
Antibacterial activity and MIC on *E. coli* and *B. subtilis* using Ciprofloxacin as a standard and antifungal activity on *C. albicans* using Clotrimazole as a standard

The free ligand exhibited the highest antimicrobial potency (the lowest MIC) followed by the Cd^2+^ then Co^2+^ complex whereas the Cu^2+^ complex was the weakest one. This MIC trend is fully consistent with the observed inhibition zone diameters and confirms the structure activity relationship inferred from the screening assays. The findings indicate that the H_2_L ligand and its Cu^2+^, Co^2+^ and Cd^2+^ chelates showed modest antibacterial activity against *E. coli* with AI values of 53.8%, 19.2%, 38.5% and 42.3%, respectively. Similarly to *B. subtilis*, the corresponding AI values compared to *ciprofloxacin* [48] were 69.6%, 30.4%, 47.8% and 60.9%. Furthermore, all the compounds showed modest antifungal properties against *C. albicans*, with AI values ​​of 44.4%, 11.1%, 33.3% and 29.6% for H_2_L, Cu^2+^, Co^2+^ and Cd^2+^ chelates, respectively, as shown in Fig. 8. The antibacterial results revealed that the free ligand exhibited the highest activity, followed by the Cd^2+^ and Co^2+^complexes, while the Cu^2+^ complexes showed relatively lower activity against when compared with the standard drug *Ciprofloxacin*, both Gram-positive and Gram-negative bacteria (*E. coli* and *Bacillus subtilis*). This trend may be attributed to the ability of the ligand and the metal ions Cd^2+^ and Co^2+^ to diffuse more easily through microbial cell walls enhancing their bio availability. In contrast, the Cu^2+^complex revealed reduced interaction with bacterial targets leading to lower activity.

As for the antifungal activity, the ligand showed the higher activity followed by Co^2+^ then Cd^2+^ and finally Cu^2+^complex when compared to *Clotrimazole* standard. The results indicate that high complex stability is not necessarily associated with higher biological activity as a weak metal ligand interaction may enhance microbial uptake and increase reactivity. It should be noted that, although the values of the antimicrobial activity are relatively small compared to the standards, the observed inhibition is still meaningful and experimentally detectable. Therefore, the activity is interpreted as a real, measurable antimicrobial impact, even if moderate intensity rather than strong action.

**DPPH method:** The antioxidant capacity of the isolated compounds was evaluated using DPPH free radical scavenging method achieved at different concentrations. The assay measures the ability of the tested compound to neutralize DPPH radicals by observing the decrease in absorbance at 517 nm using *L-ascorbic acid* as a control, as shown in Fig. 7S and Table 6S. The purple DPPH^•^ radical shows a specific absorption that decreases upon reaction with antioxidants, forming a stable yellow compound [49]. The percent of remaining DPPH^•^ was calculated using the following equations:

11 $$ \% {\text{ DPPH}}^{ \cdot } \,{\text{remaining }} = \left( {\left[ {{\mathrm{DPPH}}^{ \cdot } } \right]_{{\mathrm{t}}} / \, \left[ {{\mathrm{DPPH}}^{ \cdot } } \right]_{0} } \right) \, \times { 1}00 $$

12 $$ \% {\text{ scavenging activity }} = { 1}00 \, - \, \% {\text{ DPPH}} \cdot {\mathrm{remaining}} $$

where; T refers to the equilibrium time. The antioxidant efficiency of the tested compounds followed the order: *L-ascorbic* acid (94.5%) > H_2_L (94.0%) > Co^2+^ complex (88.8%) > Cd^2+^ complex (74.6%) > Cu^2+^ complex (62.7%). The H_2_L ligand demonstrated enriched antioxidant features similar to its metal chelates, which were due to the presence of uncoordinated functional groups, including secondary amine, azomethine, carbonyl, and pyridine nitrogen functionalities. These active centers are responsible for improving radical scavenging via hydrogen donation and electron delocalization mechanisms.

The Co^2+^ derivative had superior antioxidant capabilities due to the presence of free Schiff base nitrogen and two chloro ligands, both of which play an important part in radical neutralization, followed by Cd^2+^ chelate, which retains the uncoordinated Schiff base nitrogen atoms and a bidentate sulfato ligand, while the Cu^2+^ chelate exhibited the smallest activity due to its reliance on free pyridine nitrogen and the coordinated chloro ligand for radical interactions.

The IC_50_ value, the concentration necessary to reduce the initial DPPH^•^ concentration by 50%, was measured using the exponential curve (% DPPH^•^ remaining *versus* sample concentration (µM)). The IC_50_ values were 27.46 ± 0.18, 75.36 ± 0.47, 33.14 ± 0.21, 52.71 ± 0.29, and 16.81 ± 0.10 µM for H_2_L, Cu^2+^, Co^2+^, Cd^2+^ chelates, and *ascorbic acid*, respectively. These outcomes approve the inverse relation between IC_50_ and antioxidant efficacy, with lower IC_50_ values signifying higher antioxidant capacity [50]. Based on IC_50_ data, the antioxidant activity follows the order: *Vitamin C* > H_2_L > Co^2+^ complex > Cd^2+^ complex > Cu^2+^ complex, detecting that the free ligand exhibits superior radical scavenging ability compared to the metal complexes. Error bars represent the mean ± standard deviation in all figures of antioxidant activity (n = 3).

The (H_2_L) ligand showed the highest antioxidant impact among the compounds (IC_50_ = 27.46 ± 0.18 µM), reliable with its possession of multiple uncoordinated donor groups (-NH, azomethine, carbonyl, pyridine N), which provide flexible hydrogen-donation and electron delocalization pathways. Upon metal chelation, some of these functional groups become involved in coordination, thus reducing their availability for direct radical scavenging. Although the Co^2+^ derivative exhibited stronger activity (IC_50_ = 33.14 ± 0.21 µM) compared to the Cd^2+^ and Cu^2+^ complexes. This behavior may be associated with a combination of factors such as Co^2+^ ability to participate in redox interactions, the presence of free azomethine nitrogen capable of radical stabilization, and the influence of chloride species can effect on the electron density distribution. Nevertheless, these explanations are tentative, as DPPH^•^ reduction is multifactorial and may also be influenced by solution behavior, geometry (octahedral *vs.* distorted), ligand field effects, and steric accessibility.

The intermediate performance of the Cd^2+^ complex (IC_50_ = 52.71 ± 0.29 µM) may be attributed to its retention of uncoordinated azomethine nitrogen, while the poorer activity of the Cu^2+^ complex (IC₅₀ = 75.36 ± 0.47 µM) might relate to its stronger metal ligand binding and reduced capacity for electron transfer to stabilize DPPH^•^ radicals. However, because the antioxidant activity of Cu^2+^complexes is highly system-dependent, these interpretations cannot be considered definitive, and we have now clarified this point in the manuscript.

**Fig. 9:**
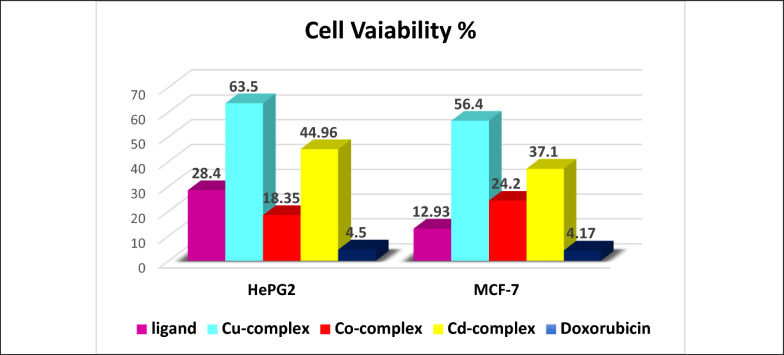
MTT cell viability percentage for the all isolated solid compounds

**Cell viability:** The cytotoxic activity of the prepared ligand and its metal chelates was estimated versus the HepG2 (human hepatocellular carcinoma) and MCF-7 (human breast cancer) cell lines via the MTT assess, as demonstrated in Fig. 9. *Doxorubicin* served as the positive control. This colorimetric technique relies on the enzymatic reduction of yellow 3-(4,5-dimethylthiazol-2-yl)-2,5-diphenyltetrazolium bromide (MTT) into insoluble purple formazan crystals by metabolically active, viable cells. The amount of formazan produced, measured spectrophotometrically, correlates directly with cell viability, which was designed according to Eq. (13) [51]:

13 $$ {\text{Cell viability }}\% \, = \, \left( {{\text{mean abs treated}}/{\text{ mean abs untreated}}} \right) \times 100 $$

Serial dilutions of each compound (100, 50, 25, 12.5, 6.25, 3.125, and 1.56 μM) were used to determine the half-maximal inhibitory concentration (IC_50_), defined as the concentration required to reduce cell viability by 50%. The obtained IC_50_ values are shown in Table 7S. Cytotoxic effects differ based on both the cell line and the compound under evaluation. The Co^2+^ complex possess the highest cytotoxic impact on the HepG2 (IC_50_ = 19.35 ± 1.4 µM). The other compounds showed weak to moderate activity with order H₂L > Cd^2+^ > Cu^2+^, where the Cu^2+^ one has the smallest cytotoxicity. The H_2_L ligand established significant anticancer properties against the MCF-7 (IC_50_ = 12.93 ± 1.1 μM) showing significant cytotoxicity. The chelates presented decreasing tendency in cytotoxicity with order Co^2+^ > Cd^2+^ > Cu^2+^.

**DNA-binding:** The DNA binding affinity of the chelates was determined via the methyl green displacement method, with *doxorubicin* serving as a positive reference. The stated outcomes are attributed to a significant reduction in methyl green absorption after interaction with the test compounds, showing efficient DNA binding. The IC_50_ values, which show the concentration required to remove 50% of the methyl green bound to DNA, are presented in Fig. 8S and given in Table 8S. The outcomes indicated the following: (i) the free ligand had the highest DNA binding affinity (IC_50_ = **29.42 ± 1.2** μM), similar to the reference drug, doxorubicin (**31.54 ± 1.5** μM), indicating a significant interaction with DNA. (ii) The Co^2+^ chelate had moderate DNA binding capacity (IC_50_ = **42.58 ± 1.8** μM), indicating a decent interaction with the DNA strand. (iii) Cd^2+^ and Cu^2+^ chelates had low DNA binding affinities (IC_50_ = **64.05 ± 3.1** μM and **76.17 ± 3.6** μM, respectively), indicating limited interaction efficiency with DNA molecules [52]. It should be noted that the methyl green displacement assay provides an indirect measure of DNA-binding affinity. Although a decrease in methyl green absorbance reflects competitive interaction with DNA, the method does not distinguish between intercalative and groove binding modes. Therefore, the present results should be interpreted as indicative of relative binding strength rather than definitive binding mechanisms.

### Molecular docking studies

**Fig. 10:**
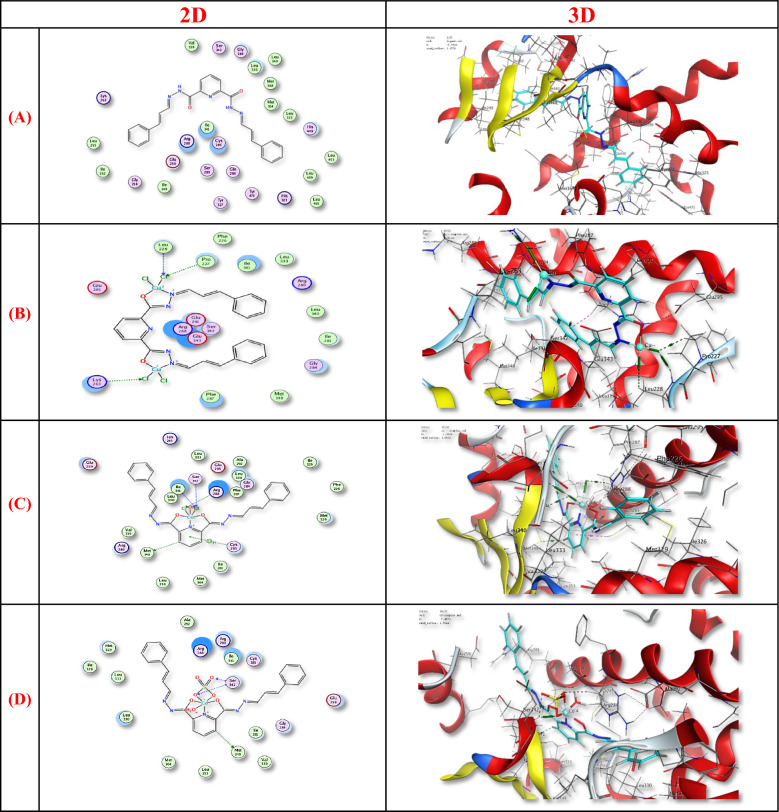
The 2D and 3D molecular docking interaction between **A** H_2_L, **B** [Cu_2_(H_2_L)Cl_4_].H_2_O, **C** [Co(H_2_L)(H_2_O)Cl_2_].3H_2_O and **D** of [Cd(H_2_L)(H_2_O)(SO_4)_].H_2_O complexes and 1i7i protien

Docking simulations were performed to estimate the binding affinity and interaction modes of the ligand and its chelates toward the 1i7i receptor. The docking parameters, including RMSD, S-scores, values, total interaction energies, and the hydrogen bonds number, are summarized in Fig. 10 and Table 4. Lower RMSD and S-scores values indicate better fitting and more stable binding poses [53, 54].

The Cd^2+^ chelate exhibited a strong binding affinity (S-score =  − 7.89 kcal/mol, RMSD = 1.53 Å, and a docking energy =  − 9.7 kcal/mol) forming three hydrogen bonds with Met348 and Ser342. The Cu^2+^ complex showed moderate binding (S =  − 6.91 kcal/mol, RMSD 1.87 Å, total energy  − 7.9 kcal/mol), stabilized by three hydrogen bonds involving Lys263, Pro227, and Leu228. The Co^2+^ chelate displayed the strongest supramolecular interaction profile (inhibition score =  − 7.11 kcal/mol, a docking energy =  − 10.6 kcal/mol, and RMSD = 1.95 Å) forming four hydrogen bonds (Met348, Arg288, Ile341, Ser342) and one π-H interaction with Cys285. These results suggest that Co^2+^ and Cd^2+^ chelates possess higher binding affinity toward the receptor compared with the Cu^2+^ chelate and the free ligand.

### Cyclic voltammetry

**Fig. 11:**
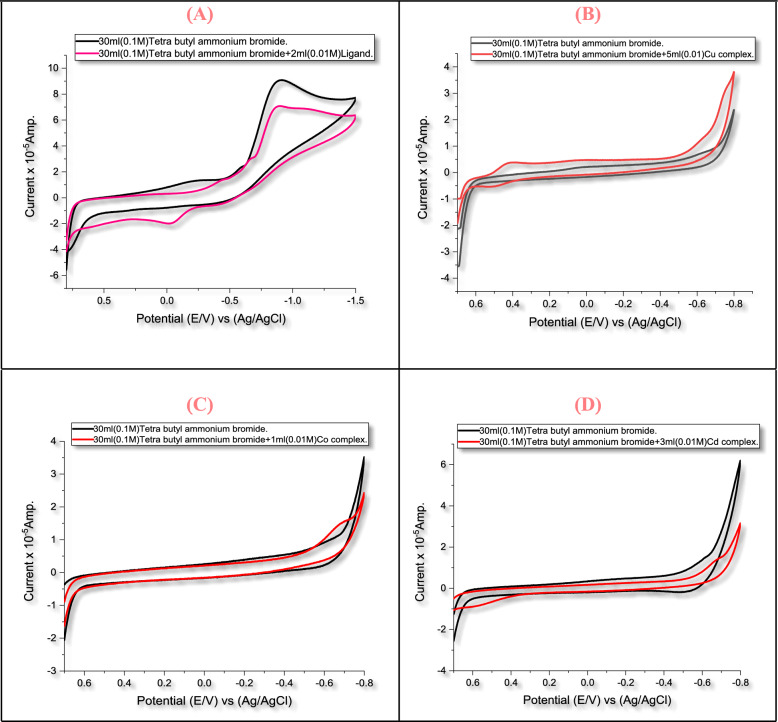
Characterization of **A** H_2_L, **B** [Cu_2_(H_2_L)Cl_4_].H_2_O, **C** [Co(H_2_L)(H_2_O)Cl_2_].3H_2_O and **D** of [Cd(H_2_L)(H_2_O)(SO_4)_].H_2_O complexes

The ligand coordination with Cu^2+^, Co^2+^, and Cd^2+^ leads to marked shifts in redox potentials and changes in reflectance patterns (Table 5). These differences reflect variations in electronic interactions between the metal and the ligand, rather than the specific oxidation–reduction mechanisms of each group individually. The electrochemical behavior of the H_2_L Schiff base was investigated in a supporting electrolyte solution of TBABr (Fig. 11A). The cyclic voltammetry shows two cathodic waves at -0.3914 V and -0.6596 V, representing stepwise ligand centered electron uptake along the extended π-conjugated structure. A broad oxidation peak was observed at -0.02763 V, indicating interweaved or quasi-reversible oxidation behavior of the ligand framework [55].

The Cu^**2+**^ complex displays a redox couple at anodic (0.5045 V) and cathodic (0.4238 V) (Fig. 11B). This performance is more reliable with a Cu^2+^/Cu^+^ redox process with possible ligand involvement. The current ratio (Ipa/Ipc = 0.4554) and peak separation (∆Ep = 0.0807) support quasi-reversible system. The voltammogram of the Co^2+^ chelate displays a single irreversible cathodic peak at  − 0.6784 V (Fig. 11C). Such irreversible conduct is representative for an EC-type mechanism associated with Co^2+^/Co^+^ reduction. The Cd^2+^ chelate shows a reduction peak at  − 0.6637 V and a broad anodic peak at 0.5851 V with a large separation (∆Ep > 0.3 V), representing irreversible ligand-centered redox processes (Fig. 11D) [56]. These electrochemical variances reveal the individual coordination environments and electronic structures forced by each metal cation.

### Structure activity relationship (SAR)

The SAR investigation of the free ligand (H_2_L) and its Cu^2+^, Co^2+^, and Cd^2+^ chelates exposes strong correlations between (structural and electronic) characteristics with the biological performance. The dipole moment and antimicrobial properties were expected by SAR where the compound becomes lipophilic when its dipole moment declines, permitting it to penetrate more efficiently through the lipid layers of microorganisms and damage them rapidly. In the current examination, the Schiff base ligand which contains uncoordinated O and N donors can provide electrons to vital receptors; its small dipole moment (6.72 De) makes it more potent against fungi and bacteria. Further, the detected modest inhibition of the chelates proposes that metal coordination confers a measurable bioactive influence compared to the free ligand consistent with literature accounts that metal identity, ligand donors, and coordination geometry strongly influence activity.

From Cell viability perspective, the global reactivity descriptors obtained from DFT investigations afford insight into the experimental cytotoxic tendencies. Though the Cu^2+^ chelate displays the lowest energy gap (E_gap_ = 0.0148 eV), representing high chemical reactivity, this doesn't interpret into improved cytotoxicity. Instead, the high reactivity may favor electronic stabilization or non-productive interactions with biomolecules, resulting in reduced biological efficiency [57].

In contrast, the free ligand exhibited fairly high cytotoxicity to both HepG2 and MCF-7 cell lines, which can be ascribed to the existence of redox active functional groups (C=N, C=O, and pyridyl N) that remain completely available in the absence of metal coordination. Metal chelation changes the electronic distribution and steric hindrance of the ligand, thus reducing its interaction with DNA and cellular goals.

## Conclusion

The synthesis and characterization of a new Schiff base ligand (H_2_L) alongside its complexes with Co^2+^, Cu^2+^, and Cd^2+^ ions, has been achieved. One of the structural results is that Cu^2+^ forms a dinuclear complex, whereas mononuclear pincer complexes of Co^2+^ and Cd^2+^ were formed. From various detailed studies using spectroscopic, thermal, and DFT methods, it can be concluded that there exist diverse coordination modes, electronic structures, and stabilities among these complexes. With the aid of DFT studies, it has been concluded that Cu^2+^ is more reactive than others because of its low value of Egab (0.0148 eV). Biologically, it has also been concluded that the free form of this ligand has very high cytotoxic activity that can surpass some of its metal chelates, whereas it also has considerable antioxidant activity. Variations in its antimicrobial, antioxidant, and cytotoxic activities disclose interesting S&A relations among these studies, in which coordination geometry, heterodonor atom coordination, and electronic density impact significantly. Docking analysis studies also showed that Cd^2+^ has considerable affinity for 1i7i receptors (Dock score:  − 9.7 kcal/mol), proposing that this compound has possible anticancer activity. In summary, the novelty of the present study lies in the diversification of Schiff base chemistry through distinct coordination modes, combined DFT-biological correlations, and the remarkable cytotoxic and docking behavior of the free ligand and its metal complexes. Future studies can include detailed mechanistic studies of its cytotoxic activity, exploration of its antimicrobial studies, and development of modified Schiff base ligands for higher selectivity.

## Supplementary Information

Additional file1 (DOCX 5557 kb)

## Data Availability

The data supporting the research findings can be obtained from the corresponding author, upon reasonable request.
