# Supplementary material for: Coordination versatility and enhanced bioactivity of Co2+, Cu2+ and Cd2+ complexes derived from pyridyl Schiff base: structural, electronic and electrochemical insights
Source: BMC Chem. 2026 Feb 21;20(1):42. doi: 10.1186/s13065-026-01730-3 (PMC12930812; doi:10.1186/s13065-026-01730-3)
Supplement: Supplementary file 1 — Additional file1 (DOCX 5557 kb) [file 13065_2026_1730_MOESM1_ESM.docx]

**Scheme 1S**: The instrument formation used for CV measurements and the shape of resulted voltammogram on computer

|  |
| --- |
| **Scheme 2S:** Antimicrobial activity screening assay |

**Scheme 3S:** Antioxidant activity for isolated compounds by DPPH method

**Scheme 4S:** The cell viability by MTT assay

|  | |
| --- | --- |
| **Scheme 5S:** colorimetric DNA-binding assay for investigated compounds |  |

| **(A)** | **** |
| --- | --- |
| **(B)** | 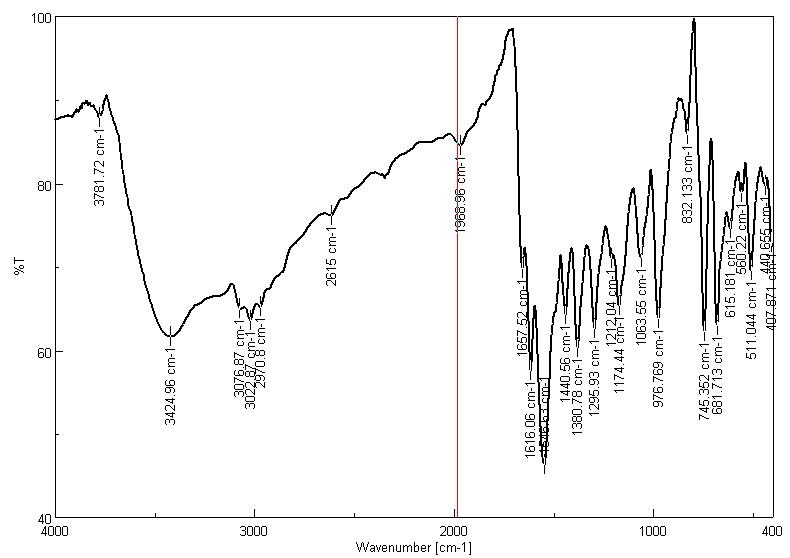 |
| **(C)** | 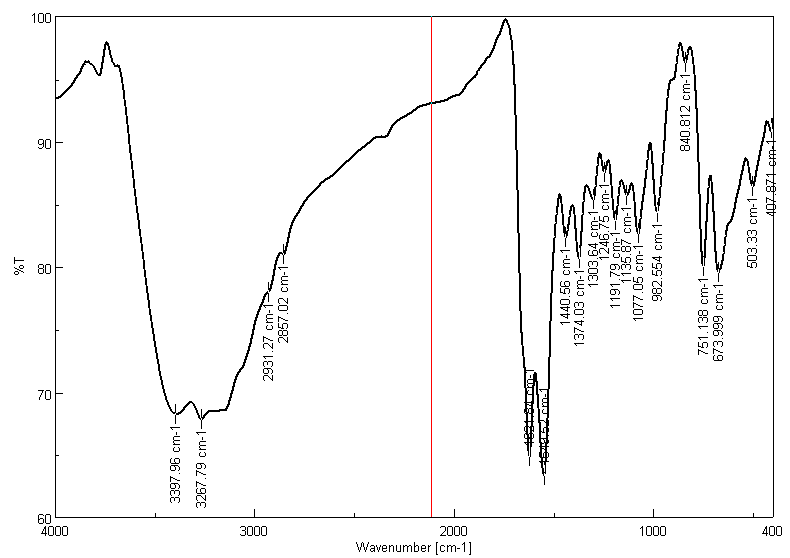 |
| **(D)** |  |
|  | **Figure 1S:** IR spectra of the (A) H_2_L ligand, (B) [Cu_2_(H_2_L)Cl_4_].H_2_O, (C) [Co(H_2_O)(H_2_L)Cl_2_].3H_2_O, and (D)[Cd(H_2_L)(H_2_O)(SO_4_)]H_2_O |

**Table 1S:** H^1^ and C^13^ NMR chemical shifts of H_2_L ligand and Cd-complex (ppm):

|  | **H^1^ NMR** | | |  | | |
| --- | --- | --- | --- | --- | --- | --- |
| Compound | δ(C_6_**H_5_**) | δ(N**H**) | δ(**HC**=CH) | δ(**H-C**=N) | δ(**C_6_**H_5_N)_Py_ | |
| H_2_L | 7.4-7.66 | 10.66 | 7.14-7.17 | 12.19 | 8.12,8.19 and 8.32 | |
| [Cd(H_2_L)(H_2_O)(SO_4_)].H_2_O | 7.28-7.35 | 11.6 | 7.057-7.11 | 12.00 | 8.19,8.42 and 8.48 | |
| **C^13^ NMR** | | | | | | |
|  | δ(**C**=N)_py_ | δ(C_6_**H_5_**) | δ(C=O) | δ(**C**=C) | δ(**C**=N) | δ(**C_5_**H_3_N)_Py_ |
| H_2_L | 148.13 | 128.93,127.28 | 159.38 | 135.81,125.6 | 139.92 | 139.92,124.82 |
| [Cd(H_2_L)(H_2_O)(SO_4_)].H_2_O | 148.9 | 127.86,126.79 | 159.06 | 129.44,125.96 | 140.67 | 129.71,116.82 |


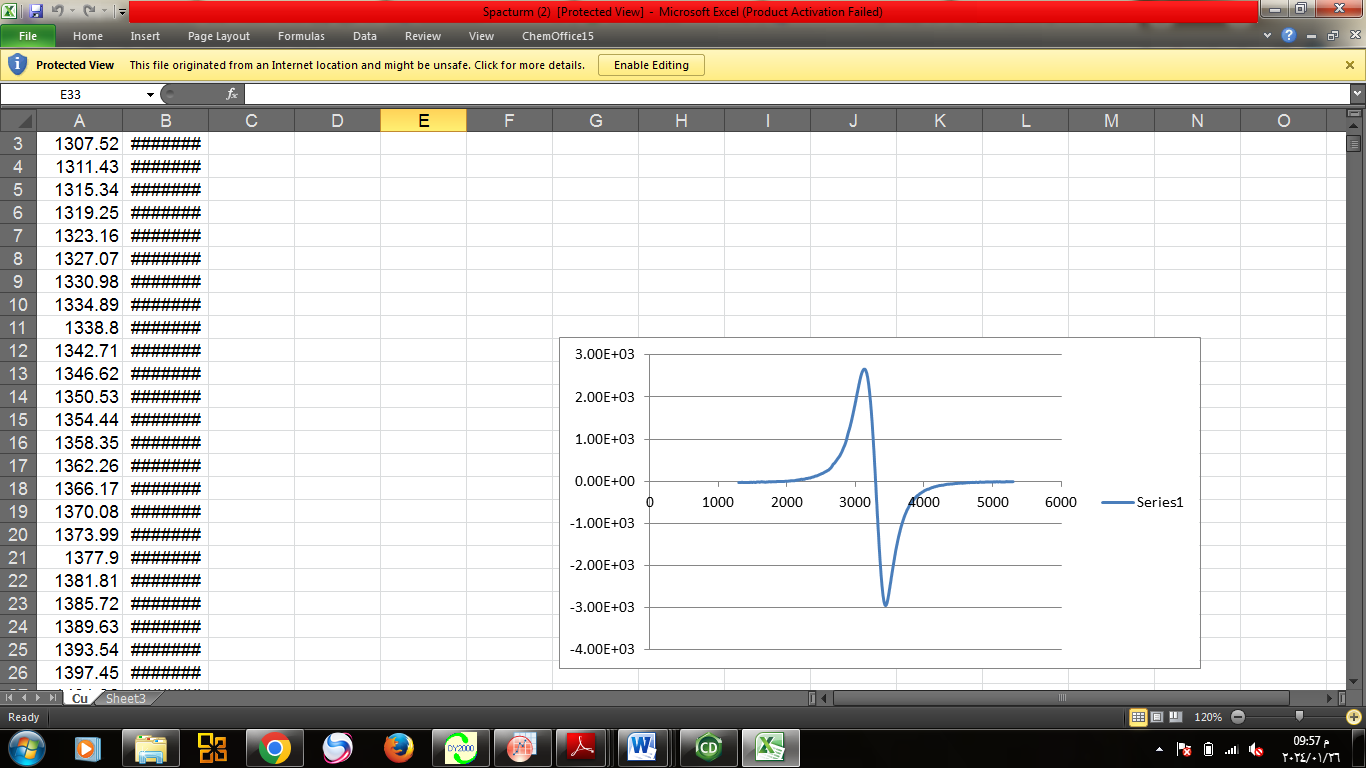


**Figure 2S:** The ESR spectrum of the [Cu_2_(H_2_L)Cl_4_].H_2_O complex

| **(A)** | 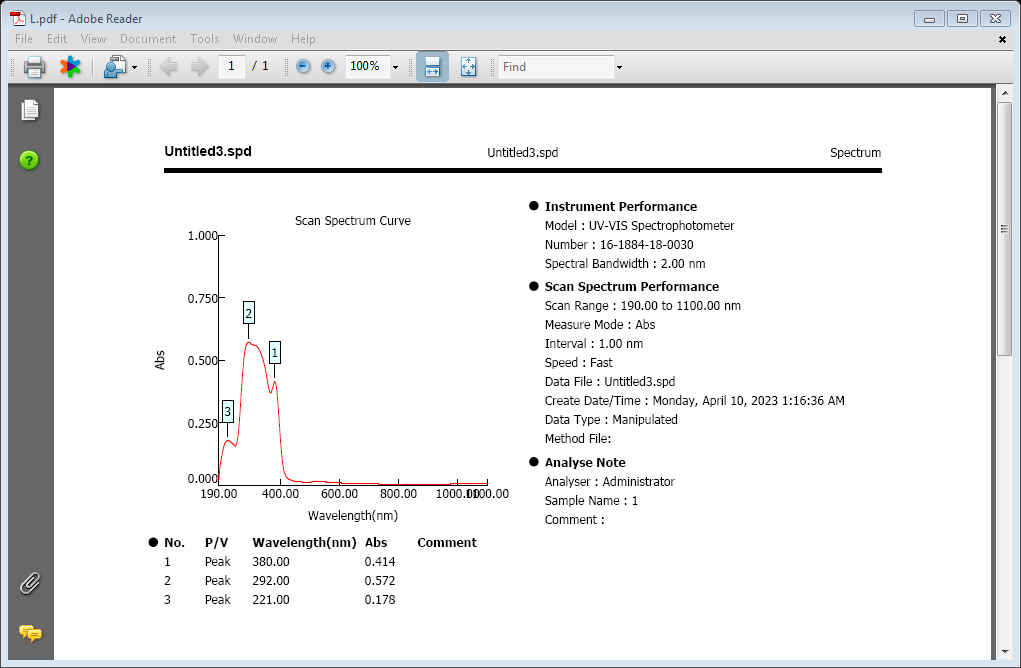 |
| --- | --- |
| **(B)** | 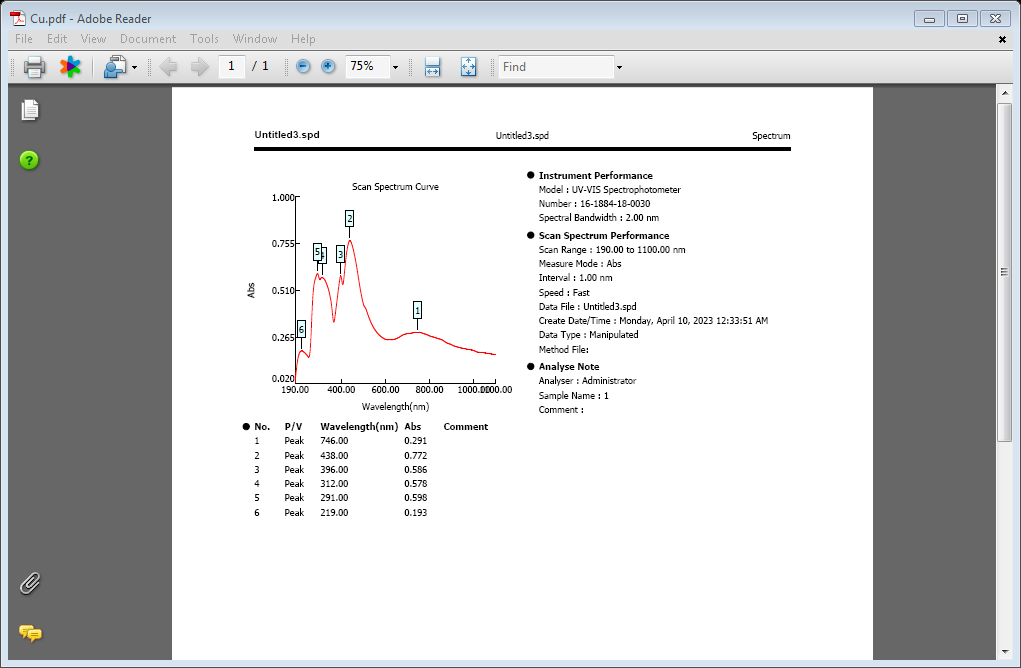 |
| **(C)** | 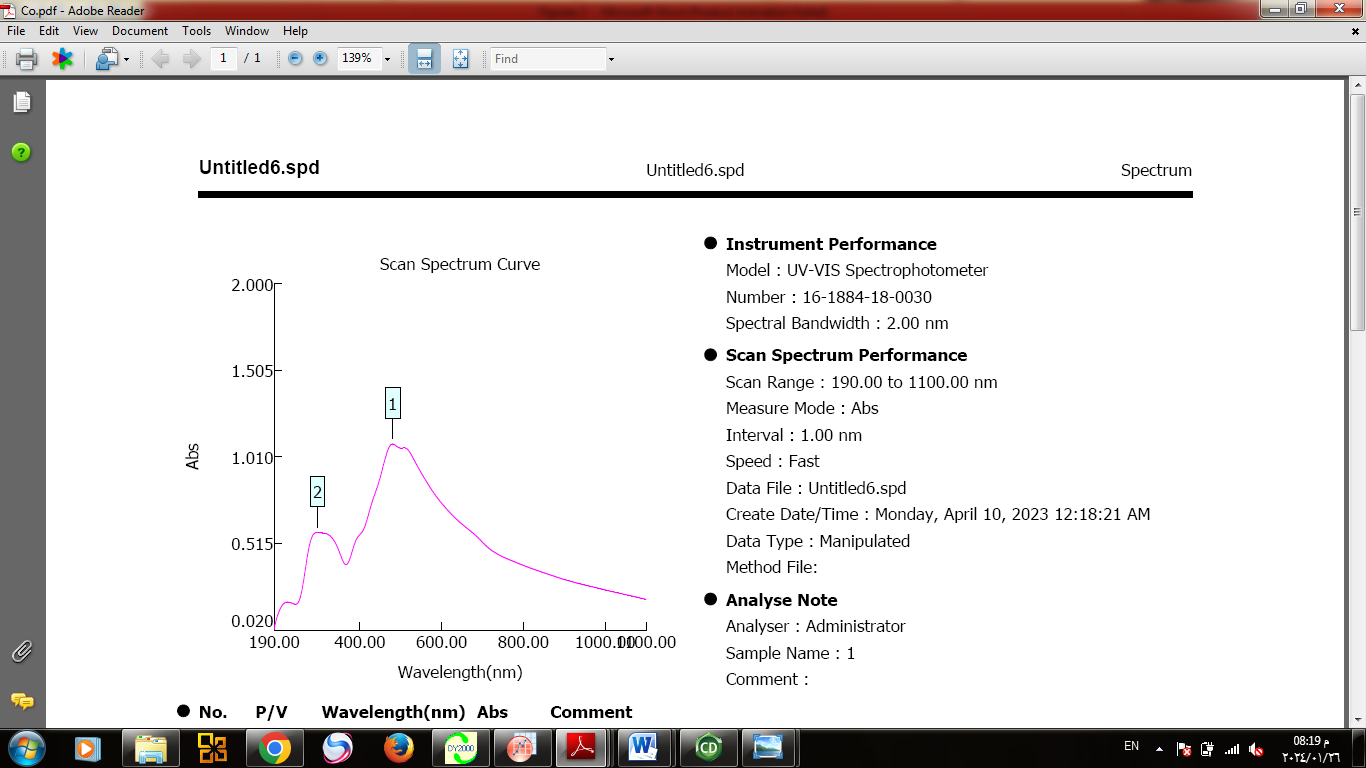 |
|  | **Figure 3S:** UV-Visible measurement of (A) H_2_L ligand, (B) [Cu_2_(H_2_L)Cl_4_].H_2_O, and (C) [Co(H_2_O)(H_2_L)Cl_2_].3H_2_O |


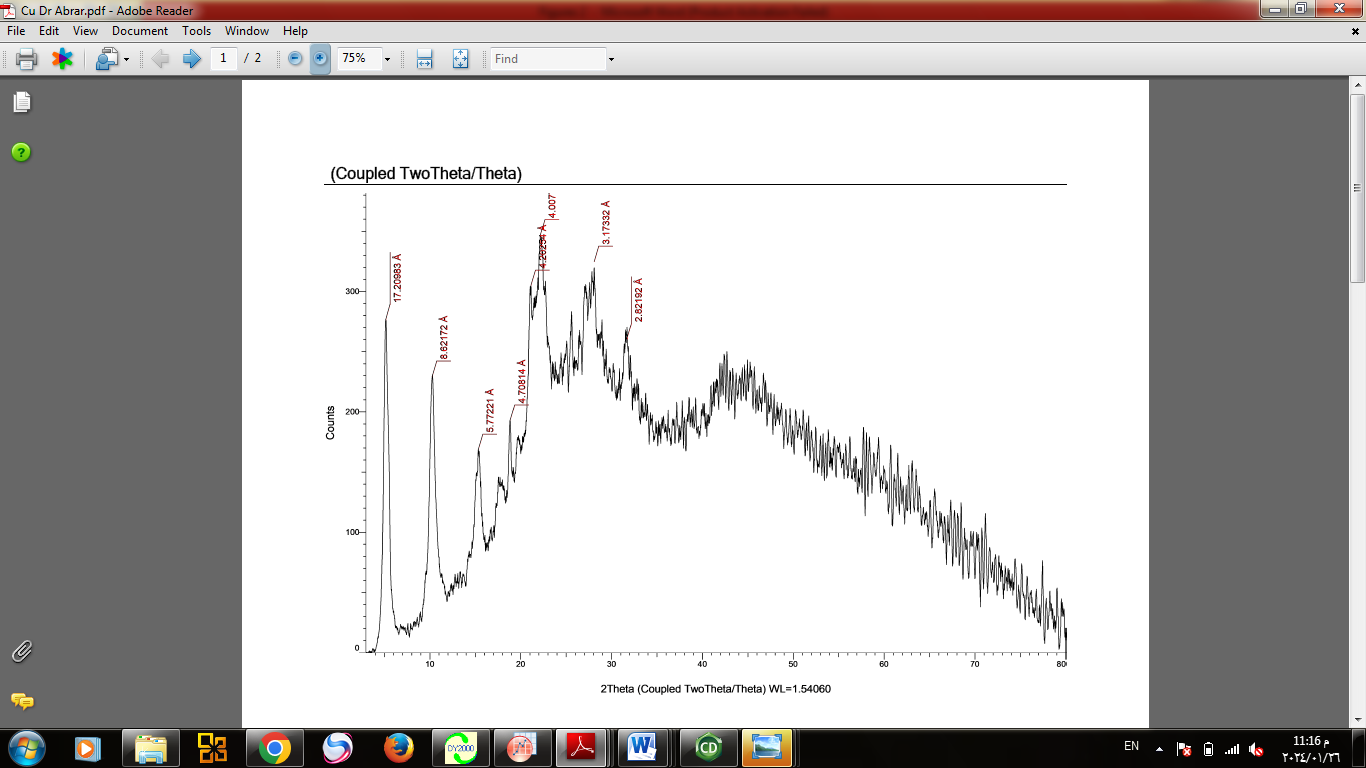


**(a)**


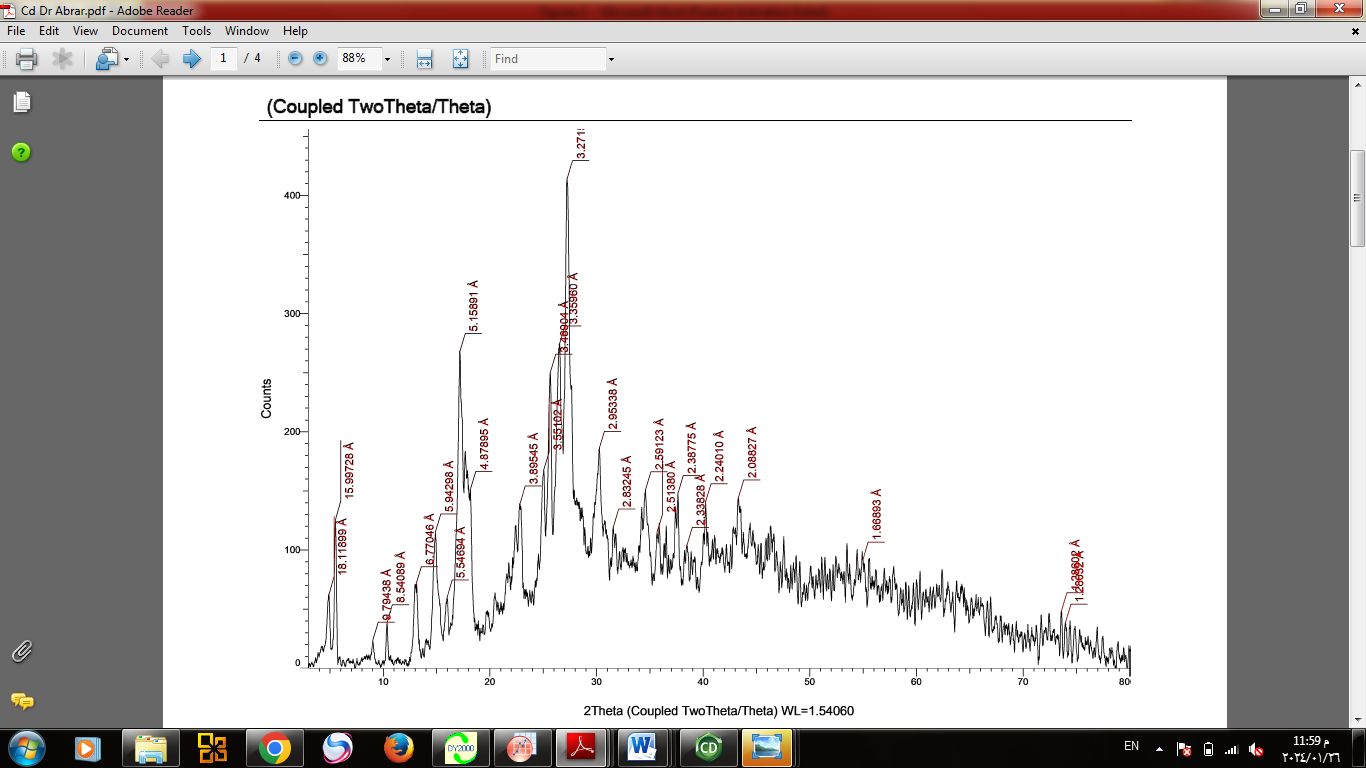


**(b)**

**Figure 4S:** PXRD of **(a)** [Cu_2_(H_2_L)Cl_4_].H_2_O complex and **(b)** [Cd(H_2_L)(H_2_O)(SO_4_)]H_2_O complex

**Table 2S:** Thermal behavior of metal complexes of H_2_L

| **Compound**  **(Molecular weight)** | **Temp. Range, (^°^C)** | **stage** | **Decomposition product(s) loss** | **Formula weight** | **Weight %**  **Found (Calcd)** |
| --- | --- | --- | --- | --- | --- |
| [Cu_2_(H_2_L)Cl_4_].H_2_O  (710.38) | 22-124 | 1^st^ | Removal of H_2_O | 18.01 | 2.20 (2.53) |
|  | 125-418 | 2^nd^ | Removal of 2Cl_2_  Removal of C_5_H_5_N | 141.80  79.10 | 32.72 (31.11) |
|  | 419-594 | 3^rd^ | Removal of 2C_6_H_6_ | 156.09 | 21.387 (21.96) |
|  | 595-665 | 4^th^ | Removal of C_8_H_4_N_4_ | 156.14 | 21.452 (22.52) |
|  | >665 | Residue | Leaving 2 CuO | 159.10 | 22.229 (22.39) |
| [Co(H_2_L)(H_2_O)Cl_2_].3H_2_O  (625.37) | 22-206 | 1^st^ | Removal of 4 H_2_O | 72.08 | 11.374 (11.5) |
|  | 207-299 | 2^nd^ | Removal of Cl_2_ | 70.90 | 12.164(11.35) |
|  | 300-416 | 3^rd^ | Removal of 2 C_9_N_2_H_9_ | 290.36 | 44.866 (46.37) |
|  | 417-470 | 4^th^ | Removal of C_7_H_3_NO | 117.10 | 19.431(18.7) |
|  | >470 | Residue | Leaving CoO | 74.93 | 12.16(11.98) |
| [Cd(H_2_L)(H_2_O)(SO_4_)]H_2_O (667.97)  250.26 | 22-207 | 1^st^ | Removal of 2H_2_O | 36.03 | 5.496 (5.39) |
|  | 208-367 | 2^nd^ | Removal of O_2_  Removal of SO_2_  Removal of 2C_6_H_5_ | 32.00  64.06  154.20 | 38.39 (37.46) |
|  | 368-538 | 3^rd^ | Removal of C_8_H_8_N_4_O | 176.17 | 25.07 (26.37) |
|  | 539-599 | 4^th^ | Removal of C_5_H_3_N | 77.08 | 12.22 (11.56) |
|  | >599 | Residue | Leaving CdO | 128.41 | 18.81 (19.22) |

|   **(a)** | **(b)** |  **(c)** |
| --- | --- | --- |

**Figure 5S:** The TG curve of the **(a)** [Cu_2_(H_2_L)Cl_4_].H_2_O **(b) [**Co(H_2_L)(H_2_O)Cl_2_].3H_2_O and **(c)** [Cd(H_2_L)(H_2_O)(SO_4_)]H_2_O complexes.

**Scheme 6S**: The outline thermal decomposition of the [Cu_2_(H_2_L)Cl_4_].H_2_O complex

**Scheme 7S**: The outline thermal decomposition of the [Co(H_2_O)(H_2_L)Cl_2_].3H_2_O complex

**Scheme 8S**: The outline thermal decomposition of the [Cd(H_2_L)(H_2_O)(SO_4_)]H_2_O complex

| **(A)** | **(B)** |
| --- | --- |
| 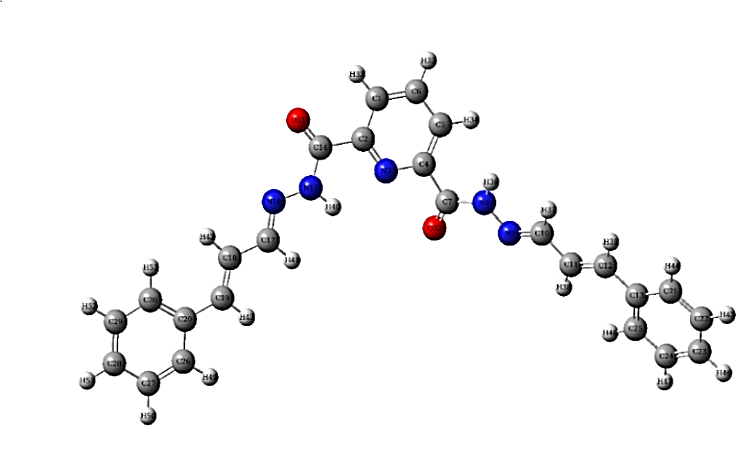 | 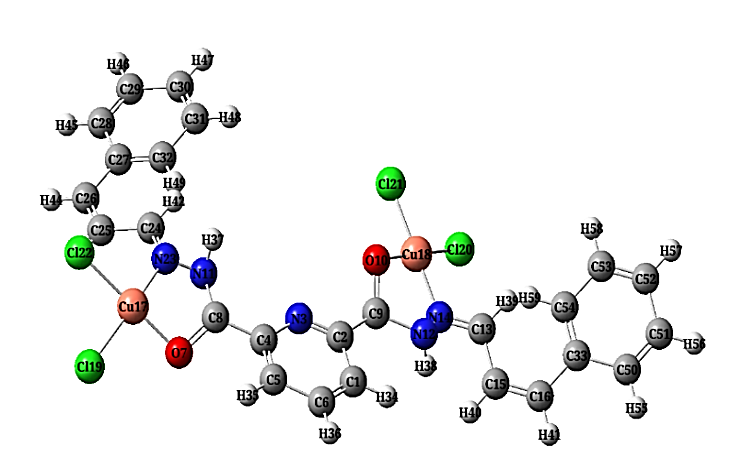 |
| **(C)** | **(D)** |
| **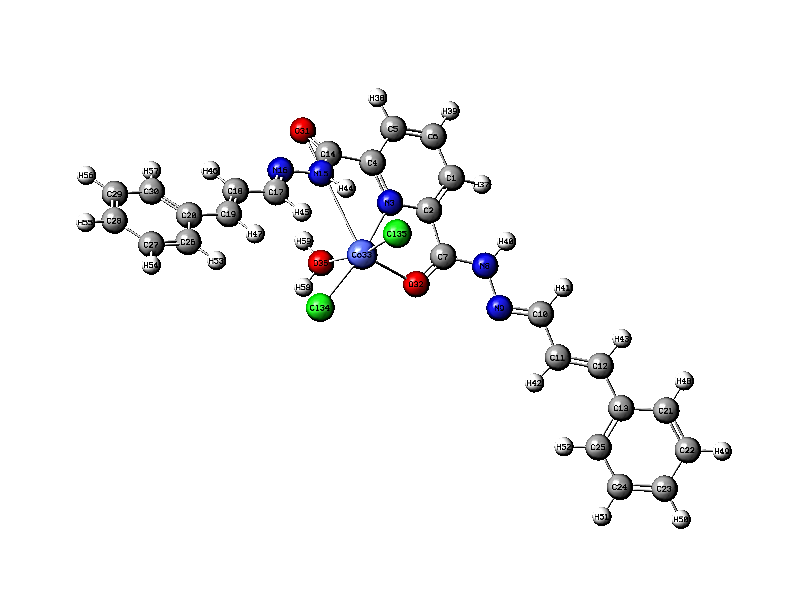** | **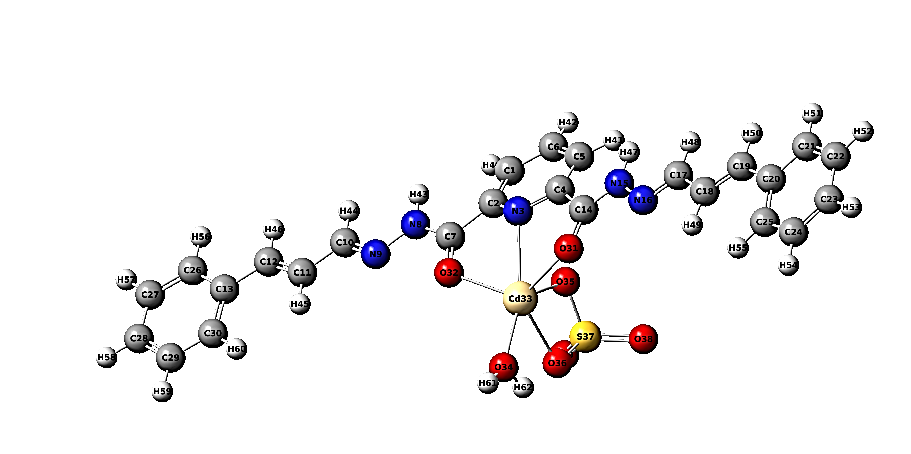** |
| **Figure 6S.** Molecular modeling for **(A)** H_2_L, **(B)** [Cu_2_(H_2_L)Cl_4_].H_2_O  **(C)** [Co(H_2_L)(H_2_O)Cl_2_].3H_2_O and **(D)** [Cd(H_2_L)(H_2_O)(SO_4_)]H_2_O complexes | |

**Table 3S.** Selected bond Lengths of H_2_L and its complexes

| **Free ligand** | | **Cu^2+^ complex** | | **Co^2+^ complex** | | **Cd^2+^ complex** | |
| --- | --- | --- | --- | --- | --- | --- | --- |
| **Bond type** | **Bond length** | **Bond type** | **Bond length** | **Bond type** | **Bond length** | **Bond type** | **Bond length** |
| N(9)-C(10) | 1.290 | O(31)-Cu(33) | 1.915 | O(36)-H(59) | 0.975 | S(37)-O(39) | 1.616 |
| N(8)-H(36) | 1.019 | N(16)-Cu(33) | 1.938 | O(36)-H(58) | 0.991 | S(37)-O(38) | 1.616 |
| C(7)-O(32) | 1.212 | O(32)-Cu(34) | 1.916 | Co(33)-O(36) | 2.033 | Cd(33)-O(36) | 2.245 |
| C(7)-N(8) | 1.388 | N(9)-Cu(34) | 1.930 | Co(33)-Cl(35) | 2.034 | Cd(33)-O(35) | 2.256 |
| C(6)-H(35) | 1.087 | Cu(33)-Cl(38) | 2.159 | Co(33)-Cl(34) | 2.302 | Cd(33)-O(34) | 2.263 |
| N(8)-N(9) | 1.358 | Cu(33)-Cl(37) | 2.159 | O(32)-Co(33) | 2.146 | O(32)-Cd(33) | 2.360 |
| N(3)-C(4) | 1.339 | Cu(34)-Cl(36) | 2.159 | O(31)-Co(33) | 2.038 | O(31)-Cd(33) | 2.360 |
| C(2)-C(14) | 1.515 | Cu(34)-Cl(35) | 2.159 | N(3)-Co(33) | 2.031 | N(3)-Cd(33) | 2.493 |
| C(2)-N(3) | 1.338 | C(7)-O(32) | 1.226 | C(29)-H(56) | 1.088 | N(3)-C(4) | 1.322 |
| C(1)-H(33) | 1.085 | C(14)-O(31) | 1.206 | N(8)-H(40) | 1.020 | O(36)-S(37) | 1.655 |

**Table 4S.** Selected bond Angles of H_2_L and its complexes

| **Free ligand** | | **Cu^2+^ complex** | | **Co^2+^ complex** | | **Cd^2+^ complex** | |
| --- | --- | --- | --- | --- | --- | --- | --- |
| **Bond type** | **Bond angle** | **Bond type** | **Bond angle** | **Bond type** | **Bond angle** | **Bond type** | **Bond angle** |
| O(31)-C(14)-N(15) | 125.774 | Cu(33)-N(16)-C(17) | 113.394 | O(36)-Co(33)-Cl(35) | 166.404 | Cd(33)-N(3)-C(4) | 111.593 |
| O(31)-C(14)-C(2) | 121.758 | Cu(33)-N(16)-N(15) | 109.628 | O(36)-Co(33)-Cl(34) | 81.899 | Cd(33)-N(3)-C(2) | 111.615 |
| N(15)-C(14)-C(2) | 112.467 | C(17)-N(16)-N(15) | 111.330 | O(36)-Co(33)-O(32) | 95.523 | O(39)-S(37)-O(38) | 116.977 |
| C(25)-C(13)-C(21) | 118.007 | H(46)-N(15)-N(16) | 121.401 | O(36)-Co(33)-O(31) | 70.976 | O(39)-S(37)-O(36) | 110.999 |
| C(25)-C(13)-C(12) | 123.228 | H(46)-N(15)-C(14) | 119.713 | O(36)-Co(33)-N(3) | 92.322 | O(39)-S(37)-O(35) | 110.266 |
| C(21)-C(13)-C(12) | 118.766 | N(16)-N(15)-C(14) | 118.883 | Cl(35)-Co(33)-Cl(34) | 96.945 | S(37)-O(36)-Cd(33) | 99.743 |
| H(37)-C(10)-C(11) | 117.860 | O(31)-C(14)-N(15) | 106.428 | Cl(35)-Co(33)-O(32) | 97.666 | S(37)-O(35)-Cd(33) | 99.261 |
| H(37)-C(10)-N(9) | 121.509 | O(31)-C(14)-C(4) | 131.663 | Cl(35)-Co(33)-O(31) | 98.176 | H(62)-O(34)-H(61) | 116.304 |
| O(32)-C(7)-N(8) | 124.292 | N(15)-C(14)-C(4) | 121.908 | Cl(35)-Co(33)-N(3) | 86.911 | H(60)-C(30)-C(29) | 119.013 |
| O(32)-C(7)-C(4) | 122.620 | Cu(34)-O(32)-C(7) | 109.016 | Cl(34)-Co(33)-O(32) | 108.589 | H(60)-C(30)-C(13) | 120.359 |

**Table 5S**: Anti-microbial assay of H_2_L ligand and its complexes

| **Compound** | ***E. coli*** | | | ***B. subtilis*** | | | ***C. Albicans*** | | |
| --- | --- | --- | --- | --- | --- | --- | --- | --- | --- |
|  | Diameter of inhibition zone  (mm) | % Activity index | MIC | Diameter of inhibition zone  (mm) | % Activity index | MIC | Diameter of  inhibition zone  (mm) | % Activity index | MIC |
| Cd-complex | 11 | 42.3 | 16 | 14 | 60.9 | 8 | 8 | 29.6 | 32 |
| Cu-complex | 5 | 19.2 | 64 | 7 | 30.4 | 32 | 3 | 11.1 | 64 |
| Ligand | 14 | 53.8 | 8 | 16 | 69.6 | 4 | 12 | 44.4 | 8 |
| Co-complex | 10 | 38.5 | 32 | 11 | 47.8 | 8 | 9 | 33.3 | 32 |
| Ciprofloxacin | 26 | 100 | 0.5 | 23 | 100 | 1 | ---- | ---- | ---- |
| Colitrimazole | ---- | ---- | ---- | ---- | ---- | ---- | 27 | 100 | 1 |

**Table 6S**: Antioxidant DPPH method on prepared compounds (Values are expressed as mean ± SD (n = 3); p < 0.05 was considered statistically significant)

| Comp. | Conc (µM) | | | | | | |
| --- | --- | --- | --- | --- | --- | --- | --- |
|  | 10 | 20 | 40 | 60 | 80 | 100 | IC50 |
|  | % Inhibition | | | | | |  |
| Vit.C | 38.7 | 52.1 | 69.6 | 81.8 | 87.4 | 94.5 | 16.81±0.10 |
| Cd-complex | 15.2 | 26.8 | 35.1 | 51.6 | 62.1 | 74.6 | 52.71±0.29 |
| Cu-complex | 9.7 | 20.3 | 29.4 | 38.9 | 51.6 | 62.7 | 75.36±0.47 |
| Ligand | 29.3 | 41.2 | 50.6 | 67.4 | 79.8 | 94.0 | 27.46±0.18 |
| Co-complex | 21.8 | 36.5 | 51.3 | 62.7 | 73.4 | 88.8 | 33.14±0.21 |

**Figure 7S:** Anti-oxidant (DPPH) method on prepared compounds using Ascorbic acid standard

**Table 7S:** Results of % cell viability and IC_50_ of (HePG2) and (MCF-7) cell lines (Values are expressed as mean ± SD (n = 3); P < 0.05 was considered statistically significant)

| **Comp.** | **In vitro Cytotoxicity** **IC_50_ (µM)*** | |
| --- | --- | --- |
|  | **HePG2** | **MCF-7** |
| **Doxorubicin** | **4.50±0.2** | **4.17±0.2** |
| **Cd** | **44.96±2.7** | **37.10±2.3** |
| **Cu** | **63.61±3.6** | **56.49±3.3** |
| **Ligand** | **28.42±2.1** | **12.93±1.1** |
| **Co** | **19.35±1.4** | **25.22±1.9** |

**IC₅₀ activity classification (very strong: 1–10 µM; strong: 11–20 µM; moderate: 21–50 µM; weak: 51–100 µM; non-cytotoxic: >100 µM) was adopted according to established guidelines reported in the literature [17].**

**Figure 8S:** DNA binding assay for prepared compounds

**Table 8S:** DNA/methyl green colorimetric assay of the compounds (Values are expressed as mean ± SD (n = 3); P < 0.05 was considered statistically significant)

| **Method** | DNA-active compound |
| --- | --- |
| **Comp.** | DNA/methyl green (IC_50_ µg/ml) |
| **DOX** | **31.54±1.5** |
| H_2_L | **29.42±1.2** |
| [Cu_2_(H_2_L)Cl_4_].H_2_O | **76.17±3.6** |
| [Co(H_2_L)(H_2_O)Cl_2_].3H_2_O | **42.58±1.8** |
| [Cd(H_2_L)(H_2_O)(SO_4_)].H_2_O | **64.05±3.1** |
